# ﻿A revision of the genus *Miccolamia* Bates from China (Coleoptera, Cerambycidae, Lamiinae, Desmiphorini)

**DOI:** 10.3897/zookeys.1264.171283

**Published:** 2025-12-11

**Authors:** Wen-Xuan Bi, Chang-Chin Chen, Mei-Ying Lin

**Affiliations:** 1 School of Life Sciences (School of Ecological Forestry) / Ecological Security and Protection Key Laboratory of Sichuan Province / Engineering Research Center for Forest and Grassland Disaster Prevention and Reduction, Mianyang Normal University, Mianyang, Sichuan 621000, China Mianyang Normal University Mianyang China; 2 Room 401, No. 2, Lane 155, Lianhua South Road, Shanghai, 201100, China Unaffiliated Shanghai China; 3 NPS office, Tianjin New Wei San Industrial Company, Ltd., Tianjin, China NPS office, Tianjin New Wei San Industrial Company, Ltd. Tianjin China; 4 State Key Laboratory of Animal Biodiversity Conservation and Integrated Pest Management, Institute of Zoology, Chinese Academy of Sciences, 1-5 Beichen West Road, Chaoyang Dist., Beijing, 100101, China Institute of Zoology, Chinese Academy of Sciences Beijing China

**Keywords:** Endophallus, longhorn beetle, new species, new synonym, Oriental region, taxonomy

## Abstract

The genus *Miccolamia* Bates, 1884 from China is revised. Sixteen species or subspecies of the genus are recognized (eight previously described and eight new): *Miccolamia savioi* Gressitt, 1940 from northwestern to eastern China, *M. mystica* Bi & Lin, **sp. nov.** from Hubei, *M. minuta* Bi & Lin, **sp. nov.** from Anhui, Shanghai, and Zhejiang, *M. yanziae* Bi & Lin, **sp. nov.** from Xizang, *M. holzschuhi* Bi & Chen, **sp. nov.** from Yunnan, *M. tonsilis* Holzschuh, 2010 from Gansu, Shaanxi, and Hubei, *M. coenosa* Holzschuh, 2010 from Shaanxi and Hubei, *M. shennong* Bi & Chen, **sp. nov.** from Hubei, *M. panda* Bi & Chen, **sp. nov.** from Sichuan, *M. dracuncula* Gressitt, 1942 from Sichuan, Chongqing, and Hubei, *M. d. orientalis* Bi & Lin, **ssp. nov.** from Zhejiang, Anhui, Hunan, and Hubei, *M. binodosa* Pic, 1935 and *M. scintillans* Holzschuh, 2010 from Yunnan, *M. liubini* Bi & Chen, **sp. nov.** from Hainan, *M. tuberculipennis* Breuning, 1947 from Fujian, Zhejiang, Hubei, and Jiangxi, and *M. castaneoverrucosa* Hayashi, 1974 from Taiwan. One new synonym, *M. bicristata* Pesarini & Sabbadini, 1997 **syn. nov.** = *M. savioi* Gressitt, 1940 is proposed. *Miccolamia albosetosa* Gressitt, 1951 from Taiwan is treated as a taxon of uncertain generic position. *Miccolamia minuta* Bi & Lin, **sp. nov.** is considered to be the smallest cerambycid beetle known in China. *Miccolamia binodosa* Pic, 1935 and *M. scintillans* Holzschuh, 2010 are newly recorded from China. Illustrations of habitus, major diagnostic features, biological notes, a distributional map as well as an identification key are provided.

## ﻿Introduction

The longhorn beetle genus *Miccolamia* Bates, 1884 was established based on three Japanese species, with *M. cleroides* Bates, 1884 subsequently designated as the type species by Breuning (1975). In his revision of the tribe Rhodopinini, Breuning (1975) proposed a subdivision of *Miccolamia* into three subgenera, whose definitions were later partially modified by Hasegawa and N. Ohbayashi (2001). Notably, the latter authors reassigned *Miccolamia* to the tribe Apodasyini. Recent molecular phylogenetic analyses by Souza et al. (2020) synonymized Rhodopinini with Lamiini. Tavakilian and Chevillotte (2025) catalogued Apodasyini as synonymous with Desmiphorini and thus placed *Miccolamia* in the latter tribe, listing 26 recognized species and subspecies distributed primarily across the Himalayas, the Indian Peninsula, East Asia, and the Indochina Peninsula.

To date, eight species of *Miccolamia* have been recorded from China: *M. savioi* Gressitt, 1940 (Shanghai), *M. dracuncula* Gressitt, 1942 (Sichuan), *M. tuberculipennis* Breuning, 1947 (Fujian), *M. albosetosa* Gressitt, 1951 and *M. castaneoverrucosa* Hayashi, 1974 (Taiwan), *M. bicristata* Pesarini & Sabbadini, 1997 (Shaanxi), *M. tonsilis* Holzschuh, 2010 (Gansu and Shaanxi), and *M. coenosa* Holzschuh, 2010 (Shaanxi and Hubei).

In this study, we revise the taxonomy of Chinese *Miccolamia* based on comprehensive morphological examination of more than 600 specimens, including type material and topotypes. Our results include the descriptions of seven new species and one new subspecies, with proposed nomenclatural changes (new synonyms and revised statuses) and new distribution records. In total, 16 species and subspecies are recognized herein. We provide an updated distributional map and a diagnostic key to facilitate species identification. Additionally, the endophallus in everted condition is documented for the first time in *Miccolamia*, although technical limitations restricted observations to *M. savioi* and *M. minuta*. Due to the discovery of morphologically intermediate species, previously proposed subgeneric classifications are not adopted in this revision.

## ﻿Materials and methods

Material is deposited in the following institutional or private collections.

**CAWW** Collection Andreas Weigel, Wernburg, Germany

**CBWX** Collection of Wen-Xuan Bi, Shanghai, China

**CCCC** Collection of Chang-Chin Chen, Tianjin, China

**CCH** Collection of Carolus Holzschuh, Villach, Austria

**CTT** Collection of Tomáš Tichý, Ostrava, Czech Republic

**EUMJ** Ehime University Museum, Matsuyama, Japan

**IZCAS** Institute of Zoology, Chinese Academy of Sciences, Beijing, China

**NHRS** Naturhistoriska Riksmuseet, Stockholm, Sweden

**OSAKA** Osaka Museum of Natural History, Osaka, Japan

**PSGC** Collection of Patrick Scott Gorring, Florida, USA

**SHEM** Shanghai Entomological Museum, Chinese Academy of Sciences, Shanghai, China

**SNUC** Insect Collection of Shanghai Normal University, Shanghai, China

**SYSU** Sun Yat-sen University, Guangzhou, China

**TARI** Taiwan Agricultural Research Institute, Taichung, Taiwan, China

Labels of the type specimens are cited verbatim: double quotation marks (“ ”) are used for a single label, a slash (/) is used to separate lines on the same label, italics indicate handwriting, notes are included in square brackets []. Chinese characters on labels of non-type specimens are translated into English.

Habitus images were taken using a Canon EOS 60D camera in conjunction with a Canon MP-E 65mm f/2.8 1–5x Macro Lens. Detailed and genital images were taken using the same camera in conjunction with a Mitutoyo M Plan APO 10x Lens attached by Micro-Tube M26. Canon MT-24EX Macro Twin Lite Flash was used as light source. CombineZP was used for image stacking. All images were edited and arranged in plates in Adobe Photoshop CS3.

Abbreviations used for morphological measurements or proportions are as follows: **AL** – antennal length; **BL** – body length, length from front of head to elytral apices; **BW** – body width, equal to the maximum elytral width (**EW**); **EL** – elytra length along suture; **PL** – pronotum length along midline.

Abbreviations used for the description of the endophallus are as follows (cf. Figs 59, 60): **APH** – apical phallomere; **BPH** – basal phallomere; **MPH** – median phallomere; **cs** – crescent-shaped sclerites; **gn** – gonopore.

The distribution map was made based on © OpenStreetMap under the Creative Commons Attribution-ShareAlike 2.0 license (CC BY-SA 2.0).

## ﻿Taxonomy

### ﻿Genus Miccolamia

Taxon classificationAnimaliaColeopteraCerambycidae

Bates, 1884

13817FB0-6CF9-57EB-A8CE-AD066B73563C

Miccolamia Bates, 1884: 253. Type species: Miccolamia cleroides Bates, 1884, designated by Breuning 1975: 52.
Miccolamia : Gressitt 1951: 518, 533; Breuning 1963: 490; Breuning 1975: 52; Tsherepanov 1991: 39; Hasegawa and N. Ohbayashi 2001: 2; Hasegawa 2007: 313, 316, 619; Lin and Yang 2019: 260.

#### Description.

Body small to minute in size (2.2–5.3 mm long), cylindrical. Head, antennae, pronotum, and elytra bearing more or less scattered, long, suberect setae (or bristles), denser on inner side of several basal flagellomeres; pronotal and elytral setae typically dark-colored, arising from deep umbilicate punctures.

Head with frons transverse, usually convex; gena inflated; vertex flattened to medially impressed, with or without a median groove; surface smooth or rough; antennal tubercles widely separated. Eyes large, moderately to deeply emarginated; lower eye lobes vertical, generally slightly longer than gena. Antennae slender to stout, usually subequal to body length; scape fusiform to clavate; pedicel distinctly longer than wide; antennomere III longer than scape, usually subequal to or slightly longer than IV, antennomeres IV–X gradually shorter.

Pronotum elongated to weakly transverse; anterior margin as wide as head, distinctly wider than posterior margin, both margins typically lined with one transverse groove (sometimes indistinct or absent); lateral tubercles variably developed, apices acute to subacute, slightly directed upward; disk smooth to rugose, weakly to strongly convex, sometimes with two or three more or less distinct tubercles (or swellings). Scutellum more or less tongue-shaped.

Elytra length usually > 2 × their maximum width, weakly to strongly dilated beyond midlength, apices separately or conjointly rounded; humeri usually broadly rounded, each bearing a variably developed small subacute to obtuse protrusion pointing posterodorsally. Each elytron usually bearing a large subbasal elongate-oval tubercle near suture (seldom it is absent), rarely additionally with several small tubercles (or swellings) scattered mainly on apical half (Figs 27–40); disk with more or less distinct setigerous punctures forming at most seven rows. Sutural and marginal costae well developed.

Legs with femora clavate; meso- and metatibiae distinctly thickened externally near apical 1/3 or 1/4, followed by a deep sinus bordered by dense long setae; protibiae with similar but weaker structure, lacking setae. Tarsi pseudotetramerous; tarsal claws appendiculate or simple (Figs 41–43).

#### Distribution.

China, Japan, India, Vietnam, Laos, Nepal, Thailand.

#### Remarks.

Hasegawa (2007) distinguished *Miccolamia* from other Japanese genera of Apodasyini (now Desmiphorini) by two key features, i.e., setose elytra and presence of an external subapical sinus on meso- and metatibiae. These characters are probably also sufficient to distinguish this genus from other potential relatives in Asia. However, the Southeast Asian genus *Phlyarus* Pascoe, 1858 also possesses these characters. Breuning (1975) attempted to separate *Miccolamia* from *Phlyarus* based on femora being “claviform” rather than “pedunculate.” Yet, detailed comparison of holotype images of several *Phlyarus* species with *Miccolamia* specimens reveals no definitive differences in leg morphology, leaving the taxonomic relationship between these genera unresolved.

Breuning (1975) initially divided *Miccolamia* into three subgenera, later modified by Hasegawa and N. Ohbayashi (2001). Therein, they defined the nominotypical subgenus by the apical half of elytra strongly dilated and each tarsal claw provided with distinct appendage (Fig. 41). In contrast, M. (Isomiccolamia) K. Ohbayashi, 1963 was characterized by having the lateral side of elytra almost parallel or slightly dilated posteriad, elytral disk with several tubercles throughout (Figs 39, 40), and tarsal claws without distinct appendage (Fig. 43). And the third subgenus, M. (Laomiccolamia) Breuning, 1975 is characterized by the elytra lacking basal callosities. However, this classification faces inconsistencies: *M. tonsilis* and *M. coenosa*, despite lacking true subbasal tubercles (Figs 32, 33), were placed in the nominate subgenus (Holzschuh, 2010). Additionally, the new species *M. liubini* sp. nov. combines diagnostic features of both *Miccolamia* and *Isomiccolamia* (Figs 38, 42), further obscuring the subgeneric boundaries.

Given these ambiguities, we refrain from applying subgeneric classification to avoid confusion. Instead, five species groups of Chinese *Miccolamia* are provisionally proposed, as outlined in the identification key.

### ﻿Key to the species of *Miccolamia* from China

**Table:** 

1	Elytral disk at most provided with one pair of subbasal tubercles	**2**
–	Elytral disk additionally provided with several small tubercles mainly on apical half (*tuberculipennis* group)	**14**
2	Elytral subbasal tubercles large and prominent	**3**
–	Elytra without tubercles, or at most slightly swollen behind base; pronotum weakly to moderately constricted at base, without distinct transverse grooves; pronotal disk without calli or swellings (*coenosa* group)	**4**
3	Pronotal disk almost glabrous, without calli or tubercles; pronotum distinctly constricted and transversely grooved at base	**7**
–	Pronotal disk almost entirely pubescent, provided with one pair of tubercles slightly before the midlength; pronotum constricted and grooved near both anterior and posterior margins (*binodosa* group)	**12**
4	Pronotal disk with the central area glabrous, without microsculpture	***M. tonsilis***
–	Pronotal disk entirely pubescent, densely punctate and sculptured throughout	**5**
5	Both pronotum and posterior portion of elytra strongly convex in lateral view	***M. panda* sp. nov.**
–	Pronotum weakly convex in lateral view; elytra almost flat along entire length	**6**
6	Antennae 0.9 × as long as body; elytra predominantly covered with light gray pubescence	***M. shennong* sp. nov.**
–	Antennae subequal to body length; elytral light-colored pubescent area and dark maculae almost equal in size	***M. coenosa***
7	Head and pronotum uniformly light-colored, usually reddish to chestnut red; pronotal disk smooth; each elytron with a light-colored nearly transverse hairy band situated in basal 2/5 (*savioi* group)	**8**
–	Head and most of pronotum blackish; pronotal disk more or less rugose; each elytron with narrow light-colored sharply arched hairy band situated near the midlength (*rugosula* group)	**11**
8	Pronotum with a transverse basal band of light-colored hairs; elytral subbasal tubercles tufted with dense setae on tips	**9**
–	Pronotum without light-colored hairy bands; elytral subbasal tubercles generally glabrous	***M. scintillans***
9	Antennae long and stout, 1.1–1.3 × as long as body; elytra shortened, ~1.9 × as long as wide; size minute, body length 2.18–3.05 mm	***M. minuta* sp. nov.**
–	Antennae subequal to or slightly shorter than body; elytra > 2.0 × their width; size relatively larger, body length 2.58–4.83 mm	**10**
10	Elytral disk sparsely punctate on basal 2/5, punctures not forming rows; each elytron with dense light-colored hairs forming a distinct transverse band near basal 2/5	***M. savioi***
–	Elytral disk with dense punctures forming several longitudinal rows at least on basal 2/5; each elytron with sparse light-colored hairs forming a vague band near basal 1/3	***M. mystica* sp. nov.**
11	Eyes deeply emarginated, upper and lower eye lobes connected by one row of ommatidia; pronotal disk coarsely rugose; dark portion of elytra delimited by light-colored hairy bands along anterior margin, surface almost glabrous	***M. yanziae* sp. nov.**
–	Eyes moderately emarginated, upper and lower eye lobes connected by two or three rows of ommatidia; pronotal disk finely rugose; dark portion of elytra with anterior parts divided by the light-colored hairy bands, surface densely and finely pubescent	***M. holzschuhi* sp. nov.**
12	Pronotal discal tubercles prominent, as high as the elytral subbasal tubercles in lateral view; pronotum and most of elytra brown to reddish brown; elytral disk without rows of punctures	***M. binodosa***
–	Pronotal discal tubercles lower than the elytral subbasal tubercles in lateral view; pronotum and most of elytra blackish; elytral disk with punctures forming several longitudinal rows on basal half	**13**
13	Pronotum with more or less numerous light-colored hairs along midline; pronotal lateral tubercles acute; elytral apices with a well-defined light-colored transverse band	***M. dracuncula***
–	Pronotum without light-colored hairs along midline; pronotal lateral tubercles obtuse; elytra becoming only vaguely lighter at apices	***M. dracuncula orientalis* ssp. nov.**
14	Tarsal claws appendiculate; pronotum constricted and grooved basally and apically; two anterior pronotal discal tubercles much more elevated than the posterior one	***M. liubini* sp. nov.**
–	Tarsal claws simple; pronotum only constricted at base, disk without distinct transverse groove; all three pronotal discal tubercles are weakly and equally elevated	**15**
15	Vertex smooth; most of elytra and femora dark brown to blackish	***M. tuberculipennis***
–	Vertex coarsely rugose-punctate at sides; most of elytra and femora light brown	***M. castaneoverrucosa***

### ﻿ Miccolamia savioi

Taxon classificationAnimaliaColeopteraCerambycidae

Gressitt, 1940

75E6C948-D837-51D6-AF74-8931EBFF131F

Figs 1, 2, 27, 41, 44, 52, 59, 61–66, Map 1 Chinese common name: 江苏小沟胫天牛

Miccolamia savioi Gressitt, 1940: 192, pl. V. fig. 2. Type locality: Zi-ka-wei (= Xujiahui), Shanghai.
Miccolamia savioi : Gressitt 1951: 533, 534; Breuning 1963: 490; Hua 2002: 216.
Miccolamia (Miccolamia) savioi : Breuning 1975: 52, 53; Lin 2015: 170, figs p. 171; Löbl and Smetana 2010: 224; Lin and Yang 2019: 261; Danilevsky 2020: 315.
Miccolamia (Miccolamia) bicristata Pesarini & Sabbadini, 1997: 96, 115, pl. III, fig. 4. Type locality: Huashan, Shaanxi. syn. nov.

#### Type material examined.

***Holotype*** (Fig. 44), • female, “KIANG SU / *Zi*-*Ka*-*Wei* / *8.VI.22* / Musée Heude”, “Zi-ka-wei / 8.6.22”, “A. SAVIO, coll.”, “*g. 53* / *unique*” [green label], “HOLOTYPE / MICCOLAMIA / SAVIOI / J.L. Gressitt” [red label] (IZCAS).

#### Additional material examined.

**Shanghai**: • 1 male, Songjiang Dist., Tianmashan, 10 m, 2012.VI.14, leg. Wen-Xuan Bi (CBWX); • 1 male, ditto except 2013.VI.16, leg. Xiao-Bin Song (CBWX); • 1 female, ditto except 2025.VI.6, leg. Wen-Xuan Bi (CBWX); • 1 male, Humin Rd., Dianpuhe Bridge, 2014.IV.22 em. V.2, leg. Wen-Xuan Bi (CBWX); • 29 males, 14 females, ditto except 2014.V.14 (CBWX); • 1 male, 1 female, ditto except (CCH); • 1 male, 1 female, ditto except (IZCAS); • 27 males, 30 females, ditto except 2014.V.15, leg. X.-B. Song & W.-X. Bi (CBWX); • 17 males, 17 females, ditto except 2014.V.19, leg. Xiao-Bin Song (CBWX); • 10 males, 17 females, ditto except 2014.V.20, leg. Wen-Xuan Bi (CBWX); • 28 males, 45 females, ditto except 2015.V.14 (CBWX & EUMJ); • 4 males, 4 females, ditto except 2015.V.20, leg. W.-X. Bi & N. Ohbayashi (CBWX & EUMJ); • 16 males, 25 females, ditto except 2015.V.28, leg. Wen-Xuan Bi (CBWX); • 1 male, 1 female, ditto except (SHEM); • 1 female, ditto except 2016.III.18 em. IV.30 (CBWX); • 1 male, ditto except 2017.IV.24 em. V.13 (CBWX); • 14 males, 12 females, ditto except 2017.V.13 (CBWX); • 1 female, ditto except 2020.V.7 (CBWX); • 1 male, Humin Rd., Dingan Rd., 2014.V.15, leg. Xiao-Bin Song (CBWX); • 2 males, 3 females, Jiading Dist., Liudao, 2016.VI.22–23, leg. Wen-Xuan Bi (CBWX); • 4 males, 5 females, ditto except 2025.V.30–31 (CBWX); • 1 male, 2 females, Minhang Dist., Xinzhuang, 2022.VI.17, leg. Wen-Xuan Bi (CBWX); • 2 males, 2 females, ditto except 2025.V.12 (CBWX); • 1 female, ditto except em. VI.16 (CBWX); • 2 males, 2 females, ditto except Minhang Dist., Zhujiatang, 2025.VI.5 (CBWX); • 2 females, ditto except Minhang Dist., Xingzhu Rd., 2025.VI.27 (CBWX); • 1 female, ditto except 2025.VII.20 (CBWX); • 1 male, ditto except Minhang Dist., Jinmei Rd., 2025.VII.2 (CBWX); • 1 male, 1 female, Pudong Dist., 2017.VI.10, leg. Hai Ma (CBWX); • 1 female, ditto except Pudong Dist., Gaoqiaozhen, 2019.VI.24 (CBWX); • 1 male, ditto except Cangfangcun, 2020.V.13 (CBWX); • 2 females, Baoshan Dist., 2019.V.25 (CBWX); • 1 female, ditto except Baoshan Dist., Wusongkou, 2020.VI.11 (CBWX); • 1 male, 1 female, ditto except Shuichan Rd., 2020.VI.8 (CBWX). **Henan**: • 1 male, 2 females, Xinyang, Shihe Dist., Xianshancun, 100 m, 2025.V.16, leg. Wen-Xuan Bi (CBWX); • 2 males, 1 female, ditto except Shihe Dist., Tangjiawan, 130 m, 2025.V.17 (CBWX). **Anhui**: • 2 males, 3 females, Chizhou, Meicunzhen, Likengcun, 50 m, 2023.V.8, leg. Wen-Xuan Bi (CBWX). **Jiangsu**: • 1 male, 1 female, Nanjing, Jiangxinzhou, 2012.VI.11, leg. Guo-Dong Li (CBWX). **Zhejiang**: • 1 female, Xitianmushan, 550 m, 2016.VI.2, leg. Wen-Xuan Bi (CBWX); • 3 females, ditto except Xitianmushan, Xiguan, 520 m, 2016.VI.13 (CBWX); • 1 male, 1 female, Shengzhou, Simingcun, 60 m, 2023.V.15, leg. Jin-Teng Zhao (CCCC); • 4 males, 8 females, Shaoxing, Huangzezhen, 30 m, 2023.V.17, leg. Wen-Xuan Bi (CBWX); • 1 male, 1 female, ditto except 40 m, 2023.V.17, leg. Jin-Teng Zhao (CCCC). **Hubei**: • 1 male, Yichang, Houhe, 1,350 m, 2020.V.15, leg. Wen-Xuan Bi (CBWX); • 5 males, 1 female, Fangxian, Yulingou, 1,120 m, 2020.V.30, leg. Wen-Xuan Bi (CBWX). **Shaanxi**: • 11 males, 7 females, Huayin, Huashan, 450–550 m, 2014.V.26, leg. Wen-Xuan Bi (CBWX); • 1 male, 1 female, ditto except (CCH); • 1 male, 1 female, ditto except (IZCAS); • 1 male, 2 females, ditto except 680 m, 2014.V.27 (CBWX); • 8 males, 4 females, ditto except 450 m, 2014.V.29, (CBWX); • 1 male, 1 female, Meixian, Tangyuzhen, Fengshan, 700–760 m, 2020.VI.3, leg. Wen-Xuan Bi (CBWX). **Shandong**: • 1 female, Qingdao, Laoshan, 1982.VII.8, leg. Quan-Liang Wang (IZCAS).

**Figures 1–12.**
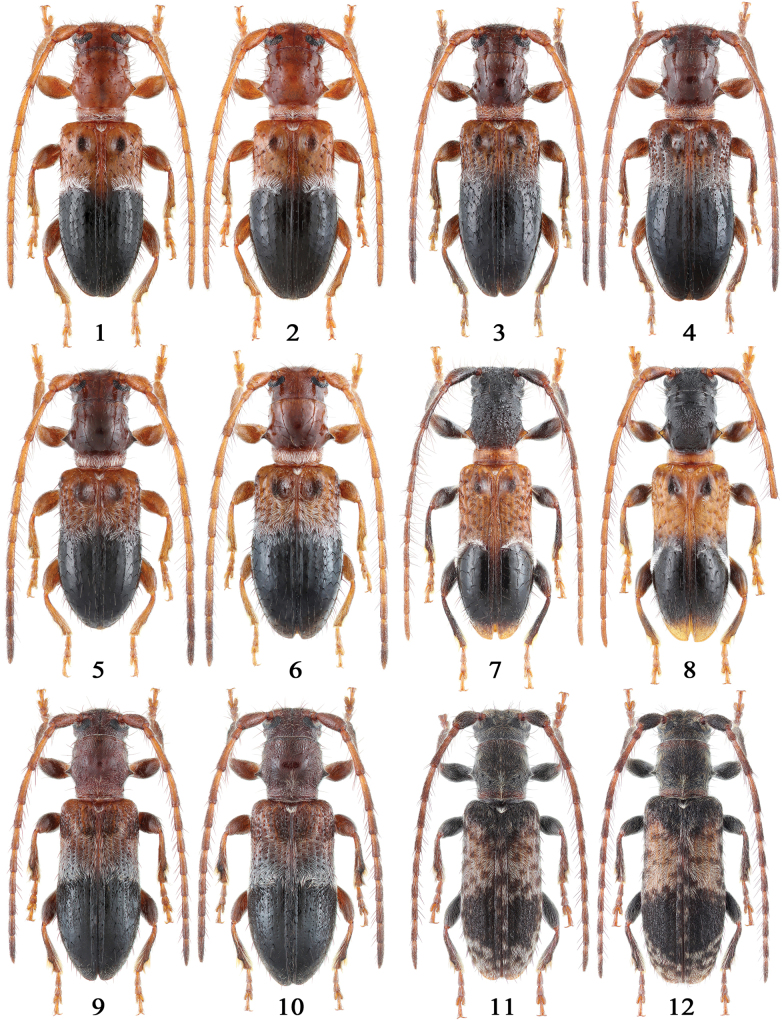
Habitus of *Miccolamia* spp. 1, 2. *M. savioi* Gressitt, 1940 from Shanghai; 3, 4. *M. mystica* sp. nov. from Hubei (3 holotype, 4 paratype); 5, 6. *M. minuta* sp. nov. from Zhejiang (5 holotype, 6 paratype); 7. *M. yanziae* sp. nov. from Xizang (holotype); 8. *M. holzschuhi* sp. nov. from Yunnan; 9, 10. *M. tonsilis* Holzschuh, 2010; 11, 12. *M. coenosa* Holzschuh, 2010; 1, 3, 5, 7, 8, 9, 11. Male; 2, 4, 6, 10, 12. Female. Not to scale.

**Map 1.**
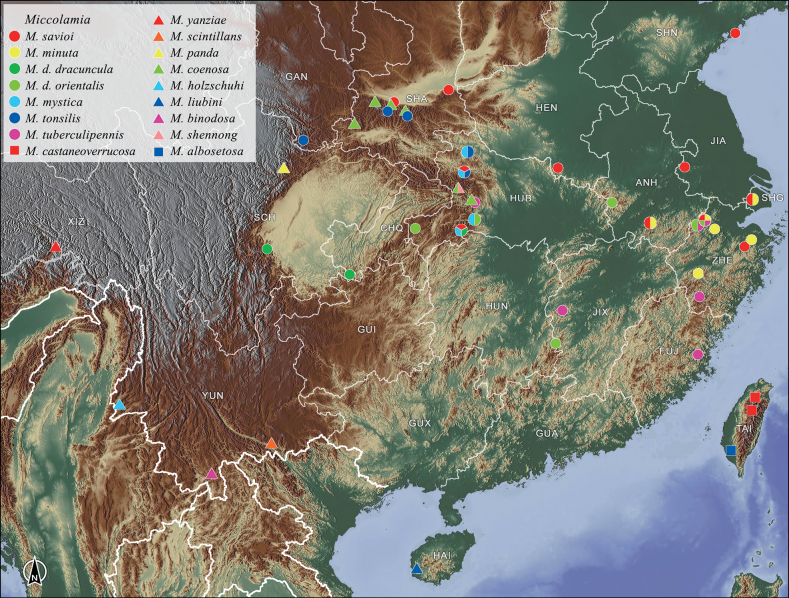
Distribution of the species of *Miccolamia* from China.

#### Other material.

• 7 later-instar larvae, 2 pupae, China, Shanghai, Humin Rd., Dianpuhe Bridge, 2015.III.21 & 29, leg. Wen-Xuan Bi (CBWX); • 1 later-instar larva, ditto (collection of Petr Švácha); • 14 later-instar larvae, ditto except 2016.III.18 (CBWX).

#### Description.

**Male** (Fig. 1). BL = 3.07–4.50, BW = 0.95–1.41 mm. Body shiny, body and appendages usually mostly reddish brown (or testaceous), head, middle portion of pronotum, scape, apical one or two antennomeres, femora and tibiae occasionally brownish; elytra typically uniformly reddish brown in basal 2/5 (rarely darkening posteriorly), remaining portion blackish; ventral surface with metaventrite brownish, abdominal ventrites dark brown to blackish. Head and appendages provided with very sparse whitish to yellowish hairs, relatively denser on frons, scape and legs, finer on apical seven antennomeres; antennomeres III-XI additionally bearing short dark brown setae throughout; pronotum with dense whitish hairs on extreme base, forming narrow transverse stripe, of which the median hairs are directed toward scutellum; scutellum covered with whitish hairs, becoming denser posteriorly; each elytron with whitish hairs forming dense transverse band occupying posterior quarter of the light-colored integument but not reaching the suture, supplemented by similar sparse hairs along basal 1/3 of suture and shorter ones near apex; ventral surface clothed with sparse yellowish hairs, denser on lateral mesoventrite, posterolateral metaventrite and posterior metanepisternum.

Head slightly wider than pronotal anterior margin, shallowly concave between antennal tubercles; frons sparsely and finely punctate. Eyes moderately emarginated, upper and lower eye lobes connected by two rows of ommatidia; lower eye lobe 1.6 × as long as wide, 1.4 × as long as gena. Antennae moderately stout, AL/BL = 1.0–1.1; scape moderately clavate, finely punctate and sculptured; antennomere III 1.1 × as long as IV, 1.4 × as long as scape, 1.6 × as long as antennomere V.

Pronotum subequal in length and width across lateral tubercles, ~1.4 × as long as basal width, constricted at basal 1/5 by a deep groove; lateral tubercles situated slightly behind midlength, moderately developed, with acute apices; disk smooth, strongly convex in lateral view (Fig. 27), very sparsely and shallowly punctate.

Elytra elongate, EL/EW = 2.0–2.1, EL/PL = 2.4–2.7, gently constricted behind obtusely angulate humeri, slightly dilated at apical 2/3, then convergent toward conjointly rounded apices; moderately convex at apical 2/3 in lateral view (Fig. 27). Each elytron provided with one large subbasal tubercle tufted with intermixed dense short and sparse long setae at tip; disk sparsely and deeply punctate; punctures gradually becoming shallower posteriorly, not forming rows (at least on basal 2/3). Ventral surface sparsely and finely punctate; abdominal ventrites finely sculptured. Legs moderately long and thick; metatibiae slightly exceeding elytral apices; tarsal claws appendiculate (Fig. 41).

***Male terminalia*.** Tergite VIII (Fig. 52a) slightly wider than long, broadly rounded apically with short fine marginal setae. Tegmen (Fig. 52b) with lateral lobe subparallel-sided, apices subacute bearing three or four long setae and few short fine setae. Median lobe (Fig. 52c) moderately curved in lateral view; apex subacute. Endophallus in everted condition (Fig. 59) S-shaped, with BPH, MPH and APH well defined; cs present; BPH moderately swollen dorsally at midlength and ventrally at apical 1/3; MPH 3 × longer than APH, constricted at extreme base, then moderately swollen forming a ventral projection pointing backwards, provided with a small sclerotized tubercle at anterior edge of the basal constriction, strongly curved dorsally near apical 1/3, with spacing a pair of small dorsal tubercles before the incurvation; APH short (possibly incompletely inflated); ejaculatory duct single; gonopore situated at apex of APH.

**Female** (Fig. 2). BL = 2.93–4.83 mm, BW = 0.99–1.52 mm. Almost identical to male in general appearance. Appendages relatively shorter. AL/BL = 1.0, EL/EW = 1.9–2.1.

#### Remarks.

This species was described by Gressitt (1940) based on a single female from Shanghai, representing the first record of the genus from China. Pesarini and Sabbadini (1997) subsequently described *M. bicristata* based on two female specimens collected at Huashan, Shaanxi, and distinguished it from *M. savioi* by its “highly raised” (vs “much smaller, rounded at the tip and moderately raised”) elytral subbasal tubercles. However, they did not examine the type material or any voucher specimens of *M. savioi*. Comprehensive comparison of both original descriptions, examination of the *M. savioi* holotype, and analysis of extensive topotypic material from both species reveal no diagnostically significant morphological differences. Consequently, *M. bicristata* Pesarini & Sabbadini, 1997 is proposed as a junior synonym of *M. savioi* Gressitt, 1940.

This species typically exhibits reddish-brown integument on the head, pronotum, and appendages, although specimens from Hubei and Zhejiang occasionally show darker brown coloration of these structures. These differences are minor and can be attributed to intraspecific variation.

#### Distribution

**(Map 1).** China: Shandong (new province record), Henan (new province record), Shaanxi (new province record), Jiangsu, Shanghai, Anhui (new province record), Zhejiang (new province record), Hubei (new province record).

### ﻿ Miccolamia mystica

Taxon classificationAnimaliaColeopteraCerambycidae

Bi & Lin sp. nov.

9790364E-E50B-5C6A-B971-5C4380FF7A60

https://zoobank.org/99F11020-CC8B-475D-9B65-150913AB0A49

Figs 3, 4, 28, 53, Map 1 Chinese common name: 迷小沟胫天牛

#### Type material.

***Holotype***: • male, “CHINA. Hubei, Shiyan / Wudangshan / 1,400 m, 2020.V.31 / leg. Wen-Xuan Bi” (SNUC). ***Paratypes*** (4 males, 4 females): • 2 females, same data as holotype (CBWX); • 1 male, same locality as holotype, “1,410 m, 2021.VI.19 / leg. Wen-Xuan Bi” (CBWX); • 1 male, “CHINA. Hubei, Yichang / Houhe / 1,350 m, 2020.V.15 / leg. Wen-Xuan Bi” (CBWX); • 1 male, “CHINA. Hubei, Fangxian / Yulingou / 1,120 m, 2020.V.30 / leg. Wen-Xuan Bi” (CBWX); • 1 female, “CHINA. Hubei, Changyang / Tianzhushan / 1,490 m, 2020.VI.28 / leg. Wen-Xuan Bi” (CBWX); • 1 male, 1 female, ditto except “1,320 m, 2021.VI.22” (CBWX).

#### Description.

**Male** (Fig. 3). BL = 2.80–3.46, BW = 0.85–1.04 mm. Body and appendages mostly reddish brown, shiny; head, pronotum, apical four or five antennomeres, and legs darker; elytra light reddish brown in basal 1/3, remaining portion blackish; ventral surface with abdominal ventrites dark brown. Head and appendages provided with very sparse whitish hairs, finer on apical three antennomeres and longer on tibiae; pronotum with similar whitish hairs, mainly scattered near anterior margin or moderately densely on basal 1/5 forming undulate band, of which the median hairs are partially directed anteriorly; scutellum covered with yellowish hairs, becoming denser posteriorly; each elytron with sparse whitish hairs forming vague transverse band extending from subbasal tubercle to near basal 1/3, not reaching suture; ventral surface clothed with yellowish hairs, sparser on abdomen and central area of metaventrite.

Head slightly wider than pronotal anterior margin, shallowly concave between antennal tubercles; frons sparsely and finely punctate, finely sculptured. Eyes moderately emarginated, upper and lower eye lobes connected by 2 rows of ommatidia; lower eye lobe 1.7 × as long as wide, 1.2 × as long as gena. Antennae moderately stout, AL/BL = 1.0–1.1; scape weakly clavate, sparsely punctate and finely sculptured; antennomere III subequal to IV, 1.4 × longer than scape or antennomere V.

Pronotum subequal in length and width across lateral tubercles, ~1.5 × as long as basal width, constricted at basal 1/5 by a shallow groove; lateral tubercles situated slightly behind the midlength, moderately developed with acute apices; disk moderately convex, very sparsely and shallowly punctate.

Elytra elongate, EL/EW = 2.0–2.2, EL/PL = 2.5–2.6, gently constricted behind the obtusely angulate humeri, weakly dilated slightly behind midlength, then convergent toward conjointly rounded apices; very weakly convex near apical 1/3 in lateral view (Fig. 28). Each elytron with single large oval-shaped subbasal tubercle tufted with long setae at tip; disk (besides those setigerous punctures) densely and deeply punctate in basal 1/3, forming six or seven longitudinal rows, abruptly becoming finer and sparser on portion of integument, almost imperceptible on apical 1/3. Ventral surface sparsely and finely punctate. Legs moderately long and thick; metatibiae barely exceeding elytral apices; tarsal claws appendiculate.

***Male terminalia*.** Tergite VIII (Fig. 53a) trapezoidal with truncate apex; apical margin bearing short fine setae. Tegmen (Fig. 53b) with lateral lobe gradually narrowed to subacute apex bearing two or three long setae and three or four short fine setae. Median lobe (Fig. 53c) moderately curved in lateral view; apex subacute.

**Female** (Fig. 4). BL = 2.58–3.77 mm, BW = 0.89–1.22 mm. Almost identical to male in general appearance. Appendages relatively shorter. AL/BL = 1.0, EL/EW = 1.9–2.0.

#### Remarks.

This new species closely resembles the comparatively widely distributed *M. savioi*, with both occurring sympatrically in Wufeng and Fang counties (Hubei Province; Map 1) and occasionally sharing the host plant *Broussonetia papyrifera* (Moraceae). However, this new species can be distinguished from *M. savioi* by the pronotal disk less convex with basal hairy band undulate (median hairs directed anteriorly); elytral dark integument relatively larger, occupying apical 2/3; elytra with basal punctures forming distinct longitudinal rows, with light-colored pubescent band broader and more vaguely defined due to being formed by relatively sparser hairs, and with subbasal tubercles tufted with only long setae.

#### Etymology.

The specific epithet is derived from the Greek *mystikós*, meaning mystical, referring to the biological and morphological situation of the new species.

#### Distribution

**(Map 1).** China: Hubei.

### ﻿ Miccolamia minuta

Taxon classificationAnimaliaColeopteraCerambycidae

Bi & Lin sp. nov.

42681D36-63C0-5D80-9E96-4203D011B67D

https://zoobank.org/ABF72ADA-E1B4-49C6-95CB-B6A56C3EC967

Figs 5, 6, 29, 54, 60, Map 1 Chinese common name: 小小沟胫天牛

#### Type material.

***Holotype***: • male, “CHINA. Zhejiang, Hangzhou / Wuchaoshan / 150–250 m, 2017.V.9–11 / leg. Wen-Xuan Bi” (SNUC). ***Paratypes*** (27 males, 30 females): • 7 males, 12 females same data as holotype (CBWX); • 1 male, 1 female same data as holotype (SHEM); • 1 male, 1 female, “CHINA. Zhejiang, Anji / Baofuzhen, Shenxicun / 400–500 m, 2016.V.12 / leg. Wen-Xuan Bi” (CBWX); • 7 males, 8 females, ditto except “220–360 m, 2016.V.21–24” (CBWX); • 5 males, 4 females, ditto except “360 m, 2016.V.31” (CBWX); • 2 males, 1 female, “CHINA. Zhejiang, Suichang / Xinluwanzhen / 240 m, 2023.V.24 / leg. Wen-Xuan Bi” (CBWX); • 1 male, “CHINA. Zhejiang, Ningbo / Longguanxiang, Wulongtan / 2009.IV.2 / leg. Jian-Qing Zhu” (CBWX); • 1 male, 2 females, “CHINA. Anhui, Chizhou / Meicunzhen, Likengcun / 50 m, 2023.V.8 / leg. Wen-Xuan Bi” (CBWX); • 1 male, 1 female, ditto except “leg. Jin-Teng Zhao” (CCCC); • 1 male, “CHINA. Shanghai, Songjiang / Dist., Dongsheshan / 50 m, 2025.VI.17 / leg. Hai Ma (CBWX).

#### Description.

**Male** (Fig. 5). BL = 2.27–3.05, BW = 0.74–0.95 mm. Body and appendages mostly reddish brown, shiny; head, pronotum and apical three or four antennomeres darker; elytra light reddish brown in basal 2/5, remaining portion blackish; ventral surface with metaventrite and abdominal ventrites light brown. Head and appendages provided with very sparse yellowish hairs, finer on apical three antennomeres and longer on tibiae; pronotum with similar yellowish hairs very sparsely scattered, and with moderately dense whitish hairs on basal 1/5 forming a transverse band, these hairs are generally directed posteriorly; scutellum covered with yellowish hairs, becoming denser posteriorly; each elytron with sparse whitish hairs forming vague transverse band extending from subbasal tubercle to slightly before midlength and reaching suture; ventral surface clothed with whitish to yellowish hairs, sparser on abdomen and the central area of metaventrite.

Head slightly wider than pronotal anterior margin, shallowly concave between antennal tubercles; frons smooth, sparsely and finely punctate. Eyes deeply emarginated, upper and lower eye lobes connected by 1–2 rows of ommatidia; lower eye lobe ~2 × as long as wide, 1.3 × as long as gena. Antennae long and stout, AL/BL = 1.2–1.3; scape moderately clavate, finely punctate; antennomere III subequal to IV, 1.4 × as long as scape, 1.3 × as long as antennomere V.

Pronotum transverse, 0.9 × as long as width across lateral tubercles, 1.3 × as long as basal width, constricted at basal 1/5 by a deep groove; lateral tubercles situated near basal 2/5, strongly developed with acute apices; disk convex, very sparsely and finely punctate.

Elytra short, EL/EW = 1.9–2.0, EL/PL = 2.3–2.4, almost parallel-sided on basal 2/5, then gently dilated at apical 2/5 before converging to conjointly rounded apices; weakly convex near apical 2/5 in lateral view (Fig. 29); humeri obtusely angulate. Each elytron provided with one large subbasal tubercle tufted with intermixed dense short and sparse long setae on tip; disk with large punctures vaguely forming five or six longitudinal rows in basal 2/5, abruptly becoming finer and sparser on dark portion. Ventral surface sparsely and finely punctate. Legs moderately long and thick; metatibiae slightly exceeding elytral apices; tarsal claws appendiculate.

***Male terminalia*.** Tergite VIII (Fig. 54a) transverse, slightly emarginated apically and broadly rounded at sides; apical margin bearing a few short fine setae. Tegmen (Fig. 54b) with lateral lobe subparallel-sided, apices subacute bearing few long and short setae. Median lobe (Fig. 54c) moderately curved in lateral view; apex obtuse. Endophallus in everted condition (Fig. 60) undulate; BPH, MPH and APH well defined; cs present; MPH ~2.5 × as long as BPH, gently curved near basal 1/4 ventrally and near basal 1/3 dorsally, then distinctly curved near midlength ventrally and near apical 1/3 dorsally, with a subrounded bulb slightly before the midlength dorsally; APH short (possibly incompletely inflated), with gonopore situated at apex.

**Female** (Fig. 6). BL = 2.18–3.05 mm, BW = 0.74–1.04 mm. Almost identical to male in general appearance. Body slightly stouter and appendages relatively shorter. AL/BL = 1.1–1.2. EL/EW = 1.9–2.0.

#### Remarks.

This new species is similar to *M. savioi* or *M. mystica* in general appearance. However, it can be readily recognized even among all congeners by the antennae remarkably long and stout, extending beyond elytral apices in both sexes; pronotum transverse; elytra rather short, subequal to or significantly shorter than twice their maximum width; and the subbasal tubercles of elytra comparatively large.

Prior to this study, several extremely small-sized cerambycid beetle species were known from China, e.g., *Exocentrus kentingensis* (2.5–5.5 mm; Kusama and Tahira 1978) from Taiwan, *E. tantillus* (2.4–2.8 mm; Holzschuh 2007) from Sichuan and *Gracilia minuta* (2.5–7.0 mm; Vitali 2018) from “N. China”, Henan and Shaanxi (Hua 2002). Additionally, the authors’ collections contained extremely small specimens belonging to undetermined *Exocentrus* species, with none measuring < 2.3 mm in total body length. Therefore, *Miccolamia minuta* currently represents the smallest documented cerambycid species in China, with the smallest recorded specimen (a female) measuring only 2.18 mm in length.

#### Etymology.

The specific epithet is derived from the Latin *minūtus*, meaning very small, which refers to the size of this new species.

#### Distribution

**(Map 1).** China: Shanghai, Anhui, Zhejiang.

### ﻿ Miccolamia scintillans

Taxon classificationAnimaliaColeopteraCerambycidae

Holzschuh, 2010

757052F6-09F3-57C3-8F01-2361257DD201

Fig. 47, Map 1 Chinese common name: 火花小沟胫天牛

Miccolamia scintillans Holzschuh, 2010: 204, fig. 51. Type locality: Soppong – Pai, Thailand.

#### Type material examined.

Holotype (Fig. 47): • female, “NW Thailand / 1. –6. 5. 1991 / SOPPONG–PAI 1800m / LEG. PACHOLÁTKO”; “HOLOTYPUS / Miccolamia / scintillans n.sp. / det. C. Holzschuh 2010” [red label] (CCH), two photographs examined provided by Carolus Holzschuh.

#### Additional material examined.

**Yunnan**: • Honghe, Lvshuihe, 640 m, 23°01'41"N, 103°24'13"E, 20.V.2018, leg. L.Z. Meng, cross window trap (CAWW), four photographs examined taken by Andreas Weigel in 2025.

#### Remarks.

Holzschuh (2010) distinguished this species from *M. savioi* mainly by the pronotum more conspicuously constricted anteriorly and without a white hairy band basally, pronotal disk less convex in lateral view, and the subbasal tubercles of elytra without tufts of setae on tips. The combinations of the above-mentioned characters are also sufficient to distinguish this species from all current congeners.

#### Distribution

**(Map 1).** China (new country record): Yunnan; Thailand.

### ﻿ Miccolamia yanziae

Taxon classificationAnimaliaColeopteraCerambycidae

Bi & Lin sp. nov.

C44405FA-0E6D-57A3-A46B-CEA9EED9EDD1

https://zoobank.org/173B567F-AAE0-494E-A3EE-7B85175ED6A8

Figs 7, 30, 55, Map 1 Chinese common name: 燕子小沟胫天牛

#### Type material.

***Holotype***: • male, “CHINA. Xizang / Motuo, 80K / 2,100 m 2012.VIII.9 / leg. Gan-Yan Yang” (SNUC). ***Paratypes*** (1 male, 1 female): 1 male, same data as holotype (CBWX); • 1 female, “Xizang, Motuo County, Damu Township / altitude: 1525 m / 2015.VIII.28 D2”, “29.4931°N, 95.4642°E / leg. Hong-Bin Liang”, “*Miccolamia* ♀ / *yanziae* / sp. nov / Det. M.Y. Lin 2021” (IZCAS, IOZ(E) 2118514).

#### Description.

**Male** (Fig. 7). BL = 4.39–5.25, BW = 1.23–1.49 mm. Head and pronotum mostly dark brown, pronotal basal quarter and lateral tubercles, scutellum, elytral basal half and a narrow apical band reddish brown, remaining portion of elytra blackish; antennae mostly dark brown, becoming slightly lighter toward apical segments; legs mostly dark brown, except coxae, trochanters, basal 1/3 of femora and tarsi, which are reddish brown; ventral surface mostly reddish brown, anterior margin of prosternum and abdominal ventrites dark brown, lighter on ventrite V. Head and pronotum provided with very sparse yellowish hairs, almost imperceptible; scutellum covered with dense yellowish hairs on posterior edge; each elytron with very sparse yellowish hairs scattered mainly on basal half (including suture) and near apical 1/5, slightly denser shortly behind the subbasal tubercle, and with dense suberect silvery-white hairs forming narrow, sharply arched band near the midlength, just along the anterior edge of dark but shiny portion, not reaching suture; elytral long setae mostly dark-brown to blackish except for those on basal half intermixed with yellowish; appendages clothed with fine moderately dense yellowish hairs; ventral surface covered with whitish to yellowish hairs, denser on lateral sides.

Head slightly wider than pronotal anterior margin, moderately concave between antennal tubercles; frons and vertex densely and coarsely punctate. Eyes deeply emarginated, upper and lower eye lobes connected by 1 row of ommatidia; lower eye lobe ~1.8 × as long as wide, 1.5 × as long as gena. Antennae slender, AL/BL = 1.1; scape moderately clavate, densely and finely punctate and sculptured especially on frontal surface; antennomere III slightly longer than IV, 1.2 × as long as scape, 1.5 × as long as antennomere V.

Pronotum elongate, 1.1 × as long as width across lateral tubercles, 1.6 × as long as basal width, with a transverse constriction and deep groove at base; lateral tubercles situated near basal 2/5, moderately developed with acute apices; disk strongly convex, very coarsely rugose-punctate.

Elytra elongate, EL/EW = 2.3, EL/PL = 2.4, subparallel-sided on basal 2/5, gently dilated near apical 2/5, then convergent toward conjointly rounded apices; weakly convex near apical 2/5 in lateral view (Fig. 30); humeri obtusely angulate. Each elytron provided with one large subbasal tubercle tufted with dense short setae at tip; disk with coarse sparse punctures forming irregular rows on basal half, abruptly becoming finer and sparser on dark portion, and displays dense microsculpture predominantly behind subbasal tubercle on basal half. Ventral surface sparsely and finely punctate. Legs moderately long and thick; meso- and metatibiae almost straight; metatibiae slightly exceeding elytral apices; tarsal claws appendiculate.

***Male terminalia*.** Tergite VIII (Fig. 55a) transverse, broadly rounded apically with short fine setae along apical margin. Tegmen (Fig. 55b) with lateral lobe constricted near midlength and subacute apically; apices bearing dense fine setae of moderate length. Median lobe (Fig. 55c) moderately curved in lateral view; apex subacute.

**Female.**BL = 4.63 mm, BW = 1.39 mm. Almost identical to male in general appearance. Body slightly stouter and appendages relatively shorter. AL/BL = 1.0. EL/EW = 2.1.

#### Remarks.

This new species is most similar to *M. rugosula* Holzschuh, 2003 from northern India in general habitus. However, it can be distinguished from the latter by the comparatively longer elytra, EL/EW = 2.2 versus 2.1; disk provided with dense microsculpture on basal half; dark portion of elytra shiny, lacking dense dark brown pubescence; absence of apical transverse band of white pubescence; and appendages, particularly the scape and tibiae, much darker.

#### Etymology.

The new species is dedicated to our friend Ms. Gan-Yan Yang (nicknamed Yanzi), who collected a portion of the type series.

#### Distribution

**(Map 1).** China: Xizang.

### ﻿ Miccolamia holzschuhi

Taxon classificationAnimaliaColeopteraCerambycidae

Bi & Chen sp. nov.

7FB581DB-ECCD-5F8F-89D9-88100B7DDCDC

https://zoobank.org/031236D2-EB60-4E73-96F4-177B5AE7D076

Figs 8, 31, 56, Map 1 Chinese common name: 霍氏小沟胫天牛

#### Type material.

***Holotype***: • male, “CHINA. Yunnan, Longchuan / Banggunjianshan / 1,700 m, 2018.VI.7 / leg. Wen-Xuan Bi” (SNUC).

#### Description.

**Male** (Fig. 8). BL = 5.17, BW = 1.46 mm. Coloration nearly identical to *M. yanziae*, except for the following distinguishing features: the elytral arch-shaped band of silvery-white hairs obliquely divided the anterior part of the dark portion, the dark portion appears non-shiny due to coverage of very fine dark brown hairs throughout, becoming white near its posterior edge where it vaguely forms a transverse band, and the appendages apparently lighter, with antennae almost entirely light orangish and abdominal ventrite V light reddish brown.

Structures also similar to *M. yanziae*, with the following exceptions: the head with frons moderately punctate, punctures becoming shallower and finer on the vertex. Eyes weakly emarginated, with upper and lower eye lobes connected by 2–3 rows of ommatidia; lower eye lobe ~1.6 × as long as wide, 1.8 × as long as gena. Antennae comparative stouter, antennomere III subequal to IV, 1.4 × as long as the scape, and 1.5 × as long as antennomere V.

The pronotum 1.1 × as long as width across lateral tubercles, 1.5 × as long as basal width, featuring both a shallow groove near the apical 1/5 and a deep groove near the basal 1/5; lateral tubercles situated near basal 2/5, moderately developed with acute apices; the disk strongly convex, finely rugose-punctate.

The elytra are elongate, EL/EW = 2.3, EL/PL = 2.5, subparallel-sided on basal 3/5 before converging toward conjointly rounded apices. Each elytron provided with one large subbasal tubercle, carinate on anterior half and tufted with dense short setae and a few long setae at tip; the disk shows sparse, coarse punctures at basal half that become shallower posteriorly, completely lacking microsculpture. Legs are moderately long and thick; with metatibiae slightly curved inward and extending beyond the elytral apices.

***Male terminalia*.** Tergite VIII (Fig. 56a) slightly wider than long with a subacute apex; apical margin bearing short fine setae. Tegmen (Fig. 56b) with lateral lobe gradually narrowed towards subacute apex; apices bearing a few very short setae. Median lobe (Fig. 56c) moderately curved in lateral view; apex obtuse.

**Female.** Unknown.

#### Remarks.

This new species resembles both *M. rugosula* Holzschuh, 2003 and *M. yanziae* sp. nov. by the general habitus, but can be distinguished from them by the eyes weakly emarginated, upper and lower eye lobes connected by 2–3 rows of ommatidia instead of 1 row; pronotal disk comparatively smooth, not coarsely rugose-punctate; elytra indistinctly dilated at apical 2/5; elytral arch-shaped band of silvery-white hairs dividing the anterior part of the dark portion instead of restricted to its anterior edge; the dark elytral surface covered with very fine dark brown hairs (neither thickly haired nor glabrous and shiny); appendages stouter; and metatibiae curved inward.

#### Etymology.

The new species is dedicated to Mr. Carolus Holzschuh, who kindly provided material from his collection for this study.

#### Distribution

**(Map 1).** China: Yunnan.

### ﻿ Miccolamia tonsilis

Taxon classificationAnimaliaColeopteraCerambycidae

Holzschuh, 2010

3E270587-4133-53EC-B95E-C238AF7057FF

Figs 9, 10, 32, 49, Map 1 Chinese common name: 扁桃小沟胫天牛

Miccolamia (*s. str.*) tonsilis Holzschuh, 2010: 207, fig. 53. Type locality: Wenxian, Gansu.
Miccolamia (Miccolamia) tonsilis : Lin and Yang 2019: 261; Danilevsky 2020: 315.

#### Type material examined.

Holotype (Fig. 49): • male, “CHINA; S-GANSU; /

Wenxian env.; / 18.–26.vi., 1993 / V. Beneš leg.”, “HOLOTYPUS / Miccolamia / tonsilis n.sp. / det. C. Holzschuh 2010” [red label] (CCH), two photographs examined provided by Carolus Holzschuh.

#### Additional material examined.

**Shaanxi**: • 1 male, Zhouzhi, Houzhenzi, 2,050 m, 2020.VI.12, leg. Wen-Xuan Bi (CBWX). **Hubei**: • 1 female, Fangxian, Yulingou, 1,120 m, 2020.V.30, leg. Wen-Xuan Bi (CBWX); • 3 males, 2 female, Shiyan, Wudangshan, 1,450 m, 2020.V.31, leg. Jin-Teng Zhao (CCCC); • 1 male, ditto except 1,410 m, 2021.VI.19, leg. Wen-Xuan Bi (CBWX).

#### Complementary measurements.

**Male** (Fig. 9). BL = 3.17–3.40 mm, BW = 1.02–1.06 mm. Female (Fig. 10). BL = 3.24–3.43 mm, BW = 1.06–1.10 mm.

#### Remarks.

Holzschuh (2010) differentiated this species from *M. savioi* based on the presence of rough surfaces on both the head and pronotum; shorter pronotum lacking white hairy basal band; elytral disk with much denser punctures and much smaller subbasal tubercles; and shorter antennomeres III and IV combined with the presence of whitish rings on the antennae.

#### Distribution

**(Map 1).** China: Shaanxi, Gansu, Hubei (new province record).

### ﻿ Miccolamia coenosa

Taxon classificationAnimaliaColeopteraCerambycidae

Holzschuh, 2010

3D41760F-5D8A-52E7-A560-4EFF77CF56A1

Figs 10, 11, 33, 50, 51, Map 1 Chinese common name: 污小沟胫天牛

Miccolamia (s. str.) coenosa Holzschuh, 2010: 208, fig. 54. Type locality: Qin Ling Shan, Shaanxi.
Miccolamia (Miccolamia) coenosa : Lin and Yang 2019: 260; Danilevsky 2020: 315.

#### Type material examined.

Holotype (Fig. 50): • male, “CHINA – Shaanxi prov. / 21–23 June 1998 / Qing Ling Shan mts.”, “road Baoji – Taibai vill. / pass 40 km S Baoji / Zd. Jindra lgt.”, “HOLOTYPUS / Miccolamia / coenosa n.sp. / det. C. Holzschuh 2010” [red label] (CCH), two photographs examined provided by Carolus Holzschuh; • 1 paratype (Fig. 51): “CHINA, W. Hubei, 2000–3000m / Dashennongjia mts. / 31°04'N, 110°03'E / lge. O Safránek, 21. –24.VI.2001”, “PARATYPUS / Miccolamia / coenosa n.sp. / det. C. Holzschuh 2010” [red label] (CCH), two photographs examined provided by Carolus Holzschuh.

#### Additional material examined.

**Shaanxi**: • 2 males, 2 females, Meixian, Taibaishan, 2,350 m, 2020.VI.6–7, leg. Wen-Xuan Bi (CBWX); • 3 males, 5 females, Huxian, Huashuping, 2,000–2,250 m, 2020.VI.20, leg. Wen-Xuan Bi (CBWX); • 1 male, Mianxian, Zhangjiahe, Tangjialiang, 1,900 m, 2020.VI.23, leg. Wen-Xuan Bi (CBWX). **Hubei**: • 1 female, Yichang, Dalaoling, 1,500 m, 2020.V.19, leg. Wen-Xuan Bi (CBWX); • 1 female, ditto except 2020.V.23 (CBWX); • 1 male, ditto except 2020.V.24 (CBWX); • 1 male, Shennongjia, Jinhouling, 2,520 m, 2020.V.28, leg. Wen-Xuan Bi (CBWX); • 1 male, 1 female, ditto except 2,400 m, leg. Jin-Teng Zhao (CCCC); • 1 male, 1 female, ditto except 2,450–2,300 m, 2021.V.27, leg. Wen-Xuan Bi (CBWX); • 1 male, Shennongjia, Hongping, Yanziya, 2,280 m, 2020.V.29, Jin-Teng Zhao (CCCC); • 2 males, Shennongjia, Yazikou, 1,960 m, 2021.V.26, leg. Wen-Xuan Bi (CBWX); • 1 male (?), China, Hubei, Shennongjia, Muyu, 3 km NW, 2,350 m, T. Tichý (CTT).

#### Complementary measurements.

**Male** (Fig. 11). BL = 3.27–4.70 mm, BW = 1.06–1.42 mm. **Female** (Fig. 12). BL = 3.72–5.18 mm, BW = 1.20–1.61 mm.

#### Remarks.

Holzschuh (2010) differentiated this species from *M. tonsilis* mainly by the longer lower eye lobes; scape more thickened; pronotal disk less convex and with rougher surface; elytral punctures finer; and coloration darker.

**Figures 13–24.**
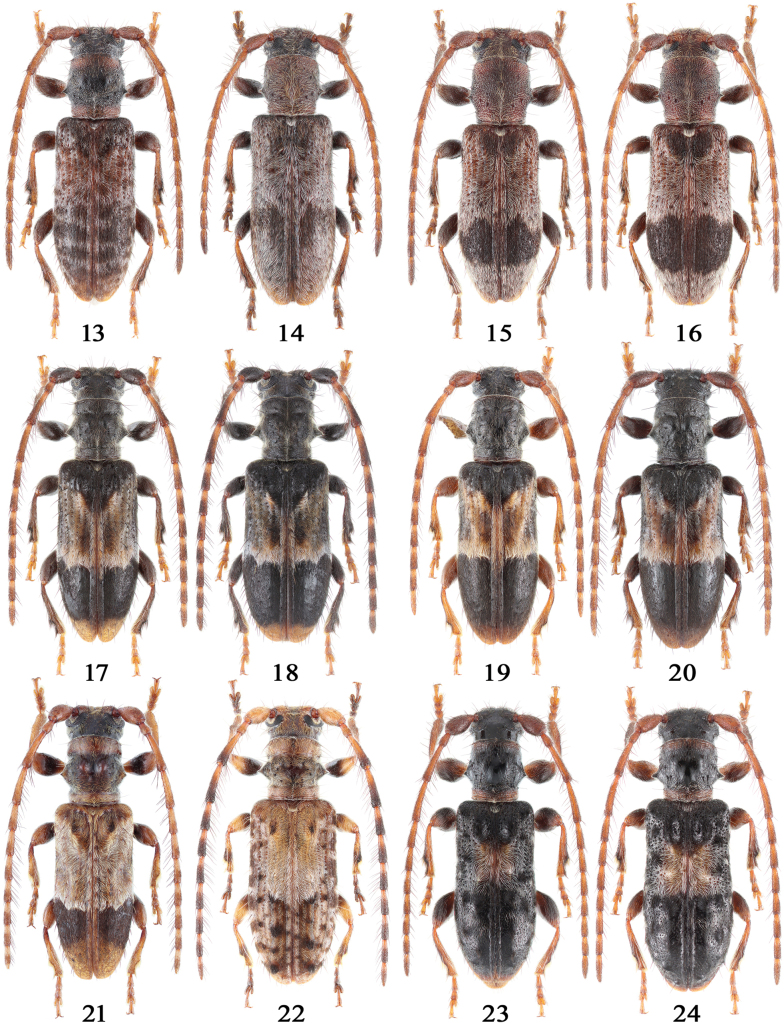
Habitus of *Miccolamia* spp.;13. *M. coenosa* Holzschuh, 2010 from Hubei; 14. *M. shennong* sp. nov. (holotype) from Hubei; 15, 16. *M. panda* sp. nov. from Sichuan (15 holotype, 16 paratype); 17, 18. *M. dracuncula* Gressitt, 1942 from Sichuan; 19, 20. *M. dracuncula orientalis* ssp. nov. from Zhejiang (19 holotype, 20 paratype); 21. *M. binodosa* Pic, 1935 from Yunnan; 22. *M. liubini* sp. nov. (holotype); 23, 24. *M. tuberculipennis* Breuning, 1947 from Zhejiang; 13–15, 17, 19, 21, 23. Male; 16, 18, 20, 22, 24. Female. Not to scale.

Some specimens from Hubei (including one paratype) slightly differ from other studied material in having the posterior dark portion of elytra interrupted by several narrow transverse stripes of light-colored pubescence (Figs 13, 51). Following Holzschuh (2010), we consider these minor variations to represent intraspecific variation rather than taxonomically significant species differences.

#### Distribution

**(Map 1).** China: Shaanxi, Hubei.

### ﻿ Miccolamia shennong

Taxon classificationAnimaliaColeopteraCerambycidae

Bi & Chen sp. nov.

E9459FC6-732B-55ED-817A-27B88FAB0EB9

https://zoobank.org/56AA1E68-9DDC-4464-B613-C8F56BCD6DDA

Figs 14, 34, 57, Map 1 Chinese common name: 神农小沟胫天牛

#### Type material.

***Holotype***: • male, “CHINA. Hubei, Shennongjia / Jinhouling / 2,520 m, 2021.V.25 / leg. Wen-Xuan Bi” (SNUC).

#### Description.

**Male** (Fig. 14). BL = 3.44, BW = 1.06 mm. Body and appendages mostly reddish brown with dull appearance; head, scape and antennomeres XI darker; elytra mostly brown with narrowly reddish brown apical margins; femora and ventral surface dark brown. Head moderately covered with yellowish to tawny pubescence; antennae covered with fine brownish pubescence intermixed with very sparse yellowish hairs, lacking light-colored rings; pronotum clothed with similar pubescence to head, except for the tawny hairs tending to form a narrow strip across the midline; scutellum densely covered with whitish hairs; elytra predominantly covered with light gray pubescence which intermixed with sparse tawny hairs mainly scattered near base or along suture, the pubescence partially interrupted by a few very small glabrous spots; disk provided with a vague patch of dark brown pubescence behind the scutellum, not exceeding basal 1/5, and with a same-colored narrow band slightly behind the midlength, distinctly broadened along the suture and directed posteriorly; legs and ventral surface moderately covered with fine yellowish pubescence throughout.

Head slightly wider than pronotal anterior margin, shallowly concave between antennal tubercles; frons densely and finely punctate and sculptured. Eyes deeply emarginated, upper and lower eye lobes connected by 1 row of ommatidia; lower eye lobe ~2 × as long as wide, 1.8 × as long as gena. Antennae short and slender, < 0.9 × body length; scape weakly clavate, finely punctate and sculptured; antennomere III subequal to IV, 1.1 × as long as scape, 1.6 × as long as antennomere V.

Pronotum ~0.9 × as long as width across lateral tubercles, 1.2 × as long as basal width; weakly constricted at base, without distinct transverse grooves; lateral tubercles small with acute apices, situated near basal 2/5; disk weakly convex, densely finely punctate and sculptured with a few interspersed large setigerous punctures.

Elytra elongate, EL/EW = 2.2, EL/PL = 3.2, gently constricted behind broadly rounded humeri, weakly dilated near apical 2/5, then convergent toward separately rounded apices; lacking subbasal tubercles but with pair of indistinct swellings in basal quarter, feebly elevated in lateral view (Fig. 34); disk sparsely and finely punctate, punctures becoming shallower toward apical 1/3, hardly forming rows. Ventral surface finely punctate. Legs moderately long and thick, metatibiae not exceeding elytral apices; femora not distinctly thickened; tarsal claws appendiculate.

***Male terminalia*.** Tergite VIII (Fig. 57a) transverse, slightly emarginated apically with broadly rounded sides; apical margin bearing few moderately long setae mainly at sides. Tegmen (Fig. 57b) with lateral lobe distinctly broadened subapically with rounded apex; bearing a few very long setae at apices and some short fine setae mainly on apical half ventrally. Median lobe (Fig. 57c) moderately curved in lateral view; apex subacute.

**Female.** Unknown.

#### Remarks.

This new species resembles *M. coenosa* and occurs sympatrically at its type locality (Map 1). But it can be readily distinguished from the latter by the antennae being comparatively shorter, distinctly shorter than 0.9 × of body length; scape less thickened; flagellomeres without basal rings of light-colored pubescence; elytra remarkably longer in relation to the pronotal length; integument of elytra almost uniformly brown, without a light-colored portion in basal half; and elytral dark-colored maculae highly reduced, occupying less than quarter of the total discal surface.

#### Etymology.

The specific epithet, Shennong, refers to one of the legendary three emperors in Chinese mythology and culture, which is also the origin of the type locality “Shennongjia” (literally means Shennong’s ladder). It is a noun in apposition.

#### Distribution

**(Map 1).** China: Hubei.

### ﻿ Miccolamia panda

Taxon classificationAnimaliaColeopteraCerambycidae

Bi & Chen sp. nov.

5808E28B-7F6B-563D-A18E-9A9CA38E2ACE

https://zoobank.org/DAC4D3A1-998F-4F87-82EE-2551F098A93E

Figs 15, 16, 35, 58, Map 1 Chinese common name: 熊猫小沟胫天牛

#### Type material.

***Holotype***: • male, “CHINA. Sichuan, Beichuan / Xiaozhaizigou, Xialiligou / 2,045 m, 2016.VIII.14 / leg. Ying-Hui Li” (SNUC). ***Paratypes*** (3 males, 1 female): 2 males, same locality as holotype “2016.VIII.14 / leg. Ying-Hui Li” (CCCC); • 1 female, “CHINA. Sichuan, Beichuan / Xiaozhaizigou, Dahuodi / 2,045 m, 2016.VIII.16 / leg. Ying-Hui Li” (CCCC); • 1 male, “CHINA. Sichuan, Beichuan / Piankou, Zhulingou / 1,713 m, 2017.VII.10 / leg. Ying-Hui Li” (CCCC).

#### Description.

**Male** (Fig. 15). BL = 3.14–3.44, BW = 0.97–1.06 mm. Body and appendages mostly brown; antennomeres III–XI basally, apical margins of elytra, basal halves of tibiae and tarsi reddish brown; femora and ventral surface dark brown. Head sparsely covered with yellowish to tawny pubescence; antennae covered with fine brownish pubescence intermixed with very sparse yellowish hairs, the latter partially forming vague basal rings on antennomeres III–XI; pronotum clothed with intermixed brownish and yellowish pubescence, the yellowish hairs forming a narrow strip along the midline; scutellum covered with dense yellowish hairs on posterior half; elytra mostly covered with gray to yellowish pubescence, comparatively sparser on basal half, occasionally interrupted by a few small glabrous spots; disk with two transverse dark brown pubescent bands, of which a small band ~1/2 of humeral width occupying basal 1/6 and a broader one ~1/4 of elytral length situated slightly behind the midlength with a sinuate anterior margin, not reaching lateral margins; legs and ventral surface moderately covered with fine yellowish pubescence throughout.

Head slightly wider than pronotal anterior margin, shallowly concave between antennal tubercles; frons densely and finely punctate. Eyes deeply emarginated, upper and lower eye lobes connected by 1 row of ommatidia; lower eye lobe ~1.9 × as long as wide, 1.7 × as long as gena. Antennae moderately long and slender, subequal to body length; scape moderately clavate, densely and finely punctate and sculptured; antennomere III 0.9 × as long as IV, 1.2 × as long as scape, 1.3 × as long as antennomere V.

Pronotum subequal to the width across lateral tubercles, 1.4 × as long as basal width; moderately constricted at base, without distinct transverse grooves; lateral tubercles situated near basal 2/5, small with acute apices; disk strongly convex, densely finely punctate with interspersed large setigerous punctures mainly on basal half.

Elytra elongate, EL/EW = 2.1–2.2, EL/PL = 2.7–2.9, subparallel-sided on basal 1/3, distinctly dilated at apical 1/3, then convergent toward separately rounded apices; distinctly convex near apical 1/3 in lateral view (Fig. 35); humeri broadly rounded; lacking subbasal tubercles but with pair of indistinct swellings in basal 1/5; disk coarsely punctate at basal half, partially forming longitudinal rows. Ventral surface with mesosternum and metaventrite densely finely rugose-punctate; elsewhere finely punctate. Legs moderately long and thick, metatibiae almost exceeding elytral apices; femora moderately thickened; tarsal claws appendiculate.

***Male terminalia*.** Tergite VIII (Fig. 58a) transverse, moderately emarginated apically with broadly rounded sides; apical margin bearing a few short fine setae. Tegmen (Fig. 58b) with lateral lobe subparallel-sided toward subacute apex; bearing a few very long setae at apices and some short fine setae mainly on apical half ventrally. Median lobe (Fig. 58c) moderately curved in lateral view; apex subacute.

**Female** (Fig. 16). BL = 3.47 mm, BW = 1.09 mm. Almost identical to male in general appearance. Body slightly stouter and appendages relatively shorter. AL/BL = 1.0. EL/EW = 2.1.

#### Remarks.

This new species is most similar to *M. coenosa* and *M. shennong* by the general habitus, especially by the elytra lacking subbasal tubercles. However, it can be readily distinguished from them by the pronotum more elongate; elytra more strongly constricted at bases; pronotum and posterior portion of elytra strongly convex in lateral view (cf. Figs 33–35); and elytral dark maculae with sharply defined edges.

#### Etymology.

The new species is named after *Ailuropoda melanoleuca* (the Giant Panda) for their shared bicolored appearance and partial geographic distribution overlap.

#### Distribution

**(Map 1).** China: Sichuan.

### ﻿ Miccolamia binodosa

Taxon classificationAnimaliaColeopteraCerambycidae

Pic, 1935

30F9F231-BCB7-5B2E-B5D1-EE9B14166B8A

Figs 21, 36, Map 1 Chinese common name: 二结小沟胫天牛

Miccolamia binodosa Pic, 1935: 15. Type locality: Tonkin, Vietnam.
Miccolamia binodosa : Breuning 1963: 490.
Miccolamia (Miccolamia) binodosa : Breuning 1975: 52, 53.

#### Material examined.

**Yunnan**: • 2 males, 2 females, Xishuangbanna, Menglun, Lvshilin, 652 m, 2019.XI.16, 21°54'42"N, 101°16'56"E, leg. Guo Tang & Zhi-Yuan Yao (IZCAS & CBWX); • 1 male, 1 female, Xishuangbanna, Menglun, Cishenglin, 644 m, 2019.XI.20, 21°54'27"N, 101°16'45"E, leg. Guo Tang & Zhi-Yuan Yao (IZCAS).

#### Complementary measurements.

Male (Fig. 21). BL = 3.90–4.75 mm, BW = 1.17–1.44 mm. Female. BL = 3.71–4.56 mm, BW = 1.20–1.45 mm.

#### Remarks.

Breuning (1975) distinguished this species from *Miccolamia cleroides* Bates, 1884 from Japan by “the pronotum with two premedian discal tubercles, reddish except at the center, the dark brown color of the apical half of the elytra reduced to a broad postmedian transverse band.”

A photograph of one specimen from Hoa Binh (determined by Breuning; probably deposited in the Natural History Museum, Basel, Switzerland), provided by Carolus Holzschuh and examined by the third author in 2008, helps confirm the identity of the Yunnan population.

#### Distribution.

China (Map 1): Yunnan (new country record); Vietnam.

### ﻿ Miccolamia dracuncula dracuncula

Taxon classificationAnimaliaColeopteraCerambycidae

Gressitt, 1942

9D328F5A-2FDB-55B7-AC83-1AD279CD8E5C

Figs 17, 18, 37, Map 1 Chinese common name: 峨眉小沟胫天牛

Miccolamia dracuncula Gressitt, 1942: 7, pl.1, fig. 8. Type locality: Omei Shan (=Emeishan), Sichuan.
Miccolamia dracuncula : Gressitt 1951: 533, 534; Breuning 1963: 490; Hua 2002: 216.
Miccolamia (Miccolamia) dracuncula : Breuning 1975: 52, 53; Löbl and Smetana 2010: 224; Lin and Yang 2019: 261; Danilevsky 2020: 315.

#### Type material examined.

Holotype (Fig. 45): • male, “Szechuan, W. China / Omei Shan: Lung-tse / to Shin-kai-sse / 1,000–1,500 M, Aug. / 13, 1940. L. Gressitt”, “HOLOTYPE / *MICCOLAMIA* / *DRACUNCULA* / J.L.Gressitt ” [red label], “峨眉小沟胫天牛♀ / *Miccolamia* / *dracuncula* Gressitt / 鉴定人: 华立中 1982” (SYSU).

#### Additional material examined.

**Sichuan**: • 1 female, Emeishan, Jiulinggang, 1,800 m, 2017.V.30, leg. Wen-Xuan Bi (CBWX); • 1 female, ditto except Wanniancun, 1,200 m, 2017.VI.2, leg. Xiao-Dong Yang (CCCC); • 1 male, 9 females ditto except Emeishan, 1,400–1,800 m, 2018.VI.16–28, leg. Wen-Xuan Bi (CBWX); • 2 males, 1 female, ditto except 1,400, 2018.VI.27, em. VII.9–VIII.10 (CBWX). **Chongqing**: • 1 male, Jiangjin, Simianshan, Dawopu, 2021.V.2–3, leg. Mei-Ying Lin (IZCAS). **Hubei**: • 1 male, Yichang, Houhe, 2013.VIII.3, leg. Hao Huang (CCCC).

#### Description.

Male (Fig. 17). BL = 3.99–4.06, BW = 1.21–1.24 mm. Head, pronotum, elytra mostly, scape, legs (excluding tarsi) and ventral surface dark brown to blackish; antennomeres II–XI light brown on basal 1/3, brown on remainders; elytra with an indistinct brownish (or testaceous) area extending from subbasal tubercles to apical 2/5 (V-shaped anteriorly, zigzag posteriorly), and with a well-defined, light brown to orangish transverse band occupying apical 1/6; tarsi light brown. Head mostly covered with sparse dark brown pubescence, genae and lateral vertex with pale to yellowish (or silvery) pubescence; antennae mostly clothed with dark brown pubescence, with yellowish pubescence sparsely distributed ventrally on scape and forming narrow basal rings on antennomeres II–XI; pronotum covered with similar pubescence to head, lighter hairs mainly situated on lateral sides and forming narrow stripe along the midline (sometimes discontinuous); scutellum covered with yellowish hairs on posterior half; elytra mostly covered with sparse dark brown pubescence, not obscuring integument, pale to yellowish pubescence arranged on the light-colored portion (denser near margins, forming V-shaped macula anteriorly and zigzag band posteriorly), and sparse yellowish pubescence on apical 1/6; legs and ventral surface moderately covered with fine yellowish pubescence.

Head slightly wider than pronotal anterior margin, moderately concave between antennal tubercles; frons densely and finely punctate. Eyes deeply emarginated, upper and lower eye lobes connected by 1 row of ommatidia; lower eye lobe ~1.7 × as long as wide, 1.3 × as long as gena. Antennae moderately long and slender, subequal to body length; scape moderately clavate, finely punctate and sculptured; antennomere III 0.9–1.0 × as long as IV, 1.3 × as long as scape, 1.4 × as long as antennomere V.

Pronotum subequal in length and width across lateral tubercles, 1.3 × as long as basal width; distinctly constricted near apical and basal 1/4 (each with transverse groove); lateral tubercles situated slightly behind the midlength, thickened at base with short acute apices, slightly pointing backward; disk weakly convex, densely and finely punctate throughout, provided with two moderate-sized premedian tubercles and scattered large setigerous punctures mainly on middle 1/3.

Elytra elongate, EL/EW = 2.2, EL/PL = 2.9, subparallel-sided on basal 1/3, weakly dilated near apical 2/5 before converging to separately rounded apices; humeri broadly rounded. Each elytron provided with one large subbasal tubercle tufted with dense short setae at tip and a slight depression behind the subbasal tubercle closer to lateral margin; disk with deep punctures forming about six longitudinal rows on basal 3/5. Ventral surface finely punctate. Legs moderately long and thick; metatibiae hardly exceeding elytral apices; tarsal claws appendiculate.

**Female** (Fig. 18). BL = 3.70–4.74 mm, BW = 1.17–1.50 mm. Almost identical to male in general appearance. Body slightly stouter and legs relatively shorter.

#### Remarks.

Gressitt (1942) separated this species from *M. binodosa* by “the prothorax black and the elytra largely black, marked with pale before middle and at apices.” It can be further distinguished from the latter by the scape less clavate, discal tubercles of pronotum less developed and elytra with distinct longitudinal rows of deep punctures.

Hua (2002) listed Fujian as one of its distributional areas. However, this distribution record could not be verified in the present study since no citations or voucher specimens were provided therein. Lin et al. 2023 (pl. XV Fig. 4) additionally reported this species from Zhejiang without providing any specimen information. This study formally provides the corresponding voucher specimen and confirms its taxonomic assignment to the herein described *Miccolamia dracuncula orientalis* ssp. nov.

#### Distribution

**(Map 1).** China: Sichuan, Chongqing (new city record), Hubei (new province record), Fujian (?).

### ﻿ Miccolamia dracuncula orientalis

Taxon classificationAnimaliaColeopteraCerambycidae

Bi & Lin ssp. nov.

2F40314F-A8AD-5E75-8545-D620D2E7DC82

https://zoobank.org/72E15F88-9B96-4B14-AAA4-C5E256E2FC19

Figs 19, 20, Map 1 Chinese common name: 峨眉小沟胫天牛东部亚种

#### Type material.

***Holotype***: • male, “CHINA. Zhejiang, Anji / Longwangshan / 1,185 m, 2017.VII.12 / leg. Wen-Xuan Bi” (SNUC). ***Paratypes*** (5 male, 6 female): 1 male, same data as holotype (CBWX); • 1 female, “CHINA. Zhejiang / Xitianmushan / 1,300 m, 2010.VII.4 / leg. Chun-Guo Xu” (CBWX); • 1 female, “Zhejiang, Xitianmushan / Houshanmen 500 m, 1998.VII.21 Ming-Shui Zhao” (IZCAS); • 1 male, “CHINA. Zhejiang, Qingliangfeng / Longtangshan / 1,120 m, 2023.VII.11 / leg. Wen-Xuan Bi” (CBWX); • 1 male, “CHINA. Zhejiang, Changhua / Qianqingtang / 1,100 m, 2008.VIII.7 / leg. Dao-Zheng Qin” (CBWX); • 1 female, “CHINA. Zhejiang, Hangzhou / Linan, Qianqingtang / 1,100–1,140 m, 2023.VII.12 / leg. Jin-Teng Zhao” (CCCC); • 1 female, ditto except “960–1,050 m, 2023.VII.13” (CCCC); • 1 male, 1 female “CHINA. Hubei, Changyang / Tianzhushan / 940 m, 2021.VI.23 / leg. Wen-Xuan Bi” (CBWX); • 1 male, “CHINA. Anhui, Yuexi / Yaoluoping / 1,400 m, 2021.VII.5 / leg. Wen-Xuan Bi” (CBWX); • 1 female, “CHINA. Hunan, Zhuzhou / Yanling, Lishuzhou, Xiaoping / 1,100–1,640 m, 2022.VI.4 / leg. Jin-Teng Zhao” (CCCC).

#### Other material examined.

**Hubei**: • 1 male, 3 females, Lichuan, Fubaoshan, 1,230 m, 2021.V.19, leg. Jin-Teng Zhao (CCCC).

#### Description.

**Male** (Fig. 19). BL = 3.24–4.88, BW = 0.96–1.54 mm. Coloration similar to the nominate subspecies except for the integument of elytral apices becoming gradually lighter, making the apical band poorly defined; the appendages particularly the antennal flagellomeres and tibiae relatively lighter; and the pronotal disk lacking the median yellowish pubescent stripe. Structures almost identical to the nominate subspecies except for the head weakly concave between antennal tubercles; pronotal disk more sparsely pubescent and pronotal lateral tubercles with thicker bases and significantly shorter apices.

**Female** (Fig. 20). BL = 3.38–4.65 mm, BW = 1.07–1.50 mm.

#### Remarks.

This new subspecies can be distinguished from the nominotypical subspecies by the aforementioned characters.

Four specimens from Lichuan, Hubei generally match the diagnostic features of *M. d. orientalis* but possess a well-defined light-colored transverse band on the elytral apices. These specimens, representing transitional forms between both subspecies, are provisionally assigned to *M. d. orientalis* but excluded from the type series.

#### Etymology.

The specific epithet is derived from the Latin *orientālis*, meaning eastern, referring to the distribution of this new subspecies.

#### Distribution

**(Map 1).** China: Zhejiang, Anhui, Hubei, Hunan.

### ﻿ Miccolamia liubini

Taxon classificationAnimaliaColeopteraCerambycidae

Bi & Chen sp. nov.

C19656A2-33BF-5C29-B940-874ED36CB90C

https://zoobank.org/C54BC09D-EA6B-490C-AFFF-9DB3BEB8EE16

Figs 38, 42, Map 1 Chinese common name: 刘彬小沟胫天牛

#### Type material.

***Holotype***: • female, “CHINA. Hainan, Ledong / Jianfengling / 1,412 m, 2017.VII.13 / leg. Bin Liu” (SNUC).

#### Description.

Female (Fig. 22). BL = 4.62, BW = 1.52 mm. Vertex of head, apical half of antennomeres III–XI, middle 1/3 of pronotum, basal half of femora, midlength to apical 1/4 of tibiae and tarsi brown to dark brown; remaining parts orangish; elytra mostly brownish, except for light brown longitudinal stripe between subbasal tubercles on basal half; ventral surface blackish. Head moderately covered with intermixed yellowish and tawny pubescence; antennae with sparse yellowish hairs on entire scape and ringed on the bases of antennomeres III–XI; pronotum clothed with similar pubescence to head, except for glabrous inverted triangle-shaped area, ~1/3 maximum pronotal width, slightly behind midlength; scutellum densely covered with yellowish hairs on posterior half; elytra with appressed yellowish and tawny pubescence forming longitudinal band on basal half, gradually becoming lighter posteriorly, remaining area mottled with pale to yellowish pubescent maculae throughout, except for a few tufts of short suberect setae arranged on the tips of the tubercles forming blackish spots mainly on apical half, disk with the long suberect setae blackish dorsally and yellowish laterally; legs and ventral surface moderately covered with fine yellowish pubescence, relatively denser on ventral surface of femora distally.

Head slightly wider than pronotal anterior margin, shallowly concave on frons and between antennal tubercles; frons densely and finely punctate. Eyes deeply emarginated, upper and lower eye lobes connected by 1 row of ommatidia; lower eye lobe ~1.7 × as long as wide, 1.4 × as long as gena. Antennae stout, AL/BL = 1.1; scape strongly clavate, densely and finely punctate, antennomere III 0.9 × as long as IV, 1.2 × as long as scape, 1.3 × as long as antennomere V.

Pronotum 0.9 × as long as width across lateral tubercles, 1.2 × as long as basal width; distinctly constricted near apical 1/3 and basal 1/4 (each with deep groove); lateral tubercles situated near midlength, thickened at base with short acute apices; disk smooth, slightly rugose on the glabrous area, weakly convex, provided with three tubercles arranged as inverted triangle, of which the anterior two are moderately large and prominent and the posterior one is flattened and indistinct, also with scattered large setigerous punctures on middle 1/3.

Elytra elongate, EL/EW = 2.0, EL/PL = 2.9, subparallel-sided on basal 1/3, gently dilated near apical 2/5, then convergent toward conjointly rounded apices; weakly convex near apical 1/3 in lateral view (Fig. 38); humeri broadly rounded. Each elytron provided with a moderate subbasal tubercle, entirely covered with short setae, and several smaller tubercles, similarly setose, scattered mainly on apical half, approximately forming two longitudinal rows, the sutural ones larger; disk also with deep and coarse punctures forming six longitudinal rows, partially extending to apical 1/3. Ventral surface finely punctate. Legs moderately long and thick; protibiae distinctly thickened near apical 1/3 externally; metatibiae hardly exceeding elytral apices; tarsal claws appendiculate (Fig. 42).

**Male.** Unknown.

#### Remarks.

This new species can be readily recognized from the congeners by a combination of the following characters: pronotum with distinct anterior and posterior constrictions and deep grooves; pronotal disk with a pair of distinct tubercles; integument of elytra without dark-colored portion; elytral disk provided with several small additional tubercles mainly on apical half; and tarsal claws appendiculate.

Notably, this species combines two key characters previously used to define separate subgenera: multi-tuberculate elytra (characteristic of *Isomiccolamia*) and appendiculate tarsal claws (diagnostic for *Miccolamia* s. str.) (Hasegawa and Ohbayashi 2001). This challenges the current subgeneric classification, prompting us to refrain from applying subgeneric division to avoid taxonomic confusion.

#### Etymology.

The new species is named after Mr. Bin Liu, who collected the holotype.

#### Distribution

**(Map 1).** China: Hainan.

### ﻿ Miccolamia tuberculipennis

Taxon classificationAnimaliaColeopteraCerambycidae

Breuning, 1947

8BA07789-7607-5586-8593-B1DB952BEA4E

Figs 23, 24, 39, Map 1 Chinese common name: 瘤翅小沟胫天牛

Miccolamia tuberculipennis Breuning, 1947: 58. Type locality: Foochow (= Fuzhou), Fujian.
Miccolamia tuberculipennis : Gressitt 1951: 533, 534; Breuning 1963: 490; Hua 2002: 216.
Miccolamia (Miccolamia) tuberculipennis : Löbl and Smetana 2010: 224; Lin and Yang 2019: 261; Danilevsky 2020: 315.
Miccolamia (Isomiccolamia) tuberculipennis : Breuning 1975: 52, 54.

#### Type material examined.

***Holotype*** (Fig. 46): • male, “*Fo chow* / *Fokien*”, “*Miccolamia* / *tuberculipenni* / *mihi Type* / det. Breuning”, “Typus” [red label], “Naturhistoriska / Riksmuseet Stockholm / Loan no 342/97”, “5251 / E94” (JLKB000027292, NHRS), three photographs examined taken by Johannes Bergsten in 2017.

#### Additional material examined.

**Zhejiang**: • 1 male, 1 female, Longquan, Fengyangshan, Luaocun, 1,100 m, 2014.IV.28, leg. Z. Peng & X.-B. Song (CBWX); • 2 females, Anji, Baofuzhen, Chenluoshan, 790 m, 2016.V.11, leg. Wen-Xuan Bi (CBWX); • 1 female, ditto except Lidongwu, 950 m, 2016.V.16 (CBWX); • 1 male, 1 female, ditto except Anji, Longwangshan, Qianmutian, 1,250 m, 2016.V.19 (CBWX); • 1 male, ditto except 2016.V.30 (CBWX); • 1 male, 1 female, Lin’an, Shenlongchuan, 880–950 m, 2017.IV.1, leg. Wen-Xuan Bi (CBWX); • 1 female, Xitianmushan, 1,200 m, 2016.VI.5, leg. Wen-Xuan Bi (CBWX); • 1 male, ditto except 1,200–1,500 m, 2017.IV.28 (CBWX); • 1 female, ditto except 1,200–1,250 m, 2017.V.1 (CBWX); • 1 female, ditto except 1,150 m, 2017.V.5 (CBWX); • 2 males, 2 females, ditto except Xitianmushan, Xiguan, 1,100–1,200 m, 2017.IV.11 (CBWX); • 1 male, 2 females, Changhua, Qianqingtang, 1,100–1,200 m, 2018.V.18, leg. Wen-Xuan Bi (CBWX). **Jiangxi**: • 1 female, Pingxiang, Wugongshan, Yangshimu, 916 m, 2013.X.24, leg. Zhong Peng (CBWX). **Hubei**: • 1 female, Yichang, Dalaoling, 1,300 m, 2020.V.23, leg. Wen-Xuan Bi (CBWX).

#### Remarks.

This species exhibits notable variation in the presence and development of a subquadrate light macula on the basal half of the elytra. The macula is moderately developed in most specimens (Figs 23, 24), completely absent (or nearly so) in three specimens including the holotype (Fig. 46), and exceptionally enlarged in one female specimen where it occupies most of the basal elytral surface.

This species shows remarkable similarity to *Miccolamia glabricula* Bates, 1884 known from Japan, particularly in elytral and tarsal morphology (characterized by multi-tuberculate elytra and simple tarsal claws). Based on comparison of a high-quality photographic series of *M. glabricula* provided by Patrick Gorring in 2025 (PSGC), *M. tuberculipennis* can be preliminarily distinguished by the scape and femora more strongly clavate and thickened (especially in males); the pronotal disk usually glabrous on the central area; and the elytral tubercles much more developed and strongly elevated in lateral view.

#### Distribution

**(Map 1).** China: Zhejiang (new province record), Hubei (new province record), Jiangxi (new province record), Fujian.

### ﻿ Miccolamia castaneoverrucosa

Taxon classificationAnimaliaColeopteraCerambycidae

Hayashi, 1974

A4D2B357-F128-5F95-989D-95EF35353E33

**Figures 25–43.**
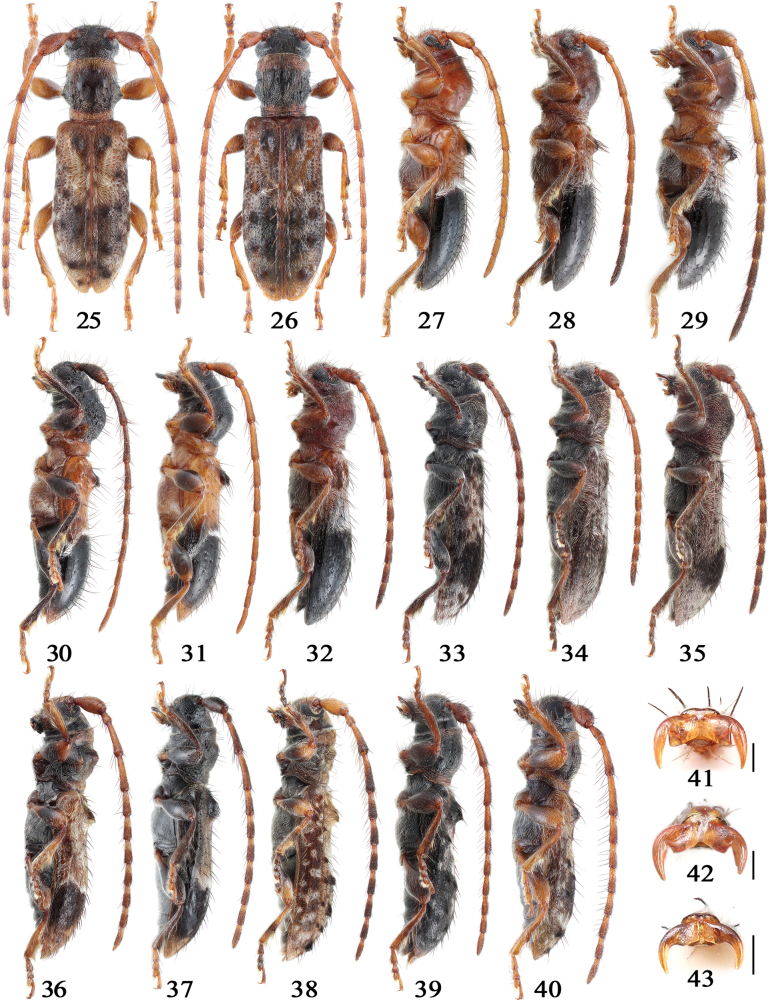
Habitus of *Miccolamia* spp. 25, 26. Dorsal view; 27–40. Lateral view; 27–40. Fore tarsal claw in frontal view; 25, 26, 40. *M. castaneoverrucosa* Hayashi, 1974 from Taiwan; 27, 41. *M. savioi* Gressitt, 1940; 28. *M. mystica* sp. nov.; 29. *M. minuta* sp. nov.; 30. *M. yanziae* sp. nov.; 31. *M. holzschuhi* sp. nov.; 32. *M. tonsilis* Holzschuh, 2010; 33. *M. coenosa* Holzschuh, 2010; 34. *M. shennong* sp. nov.; 35. *M. panda* sp. nov.; 36. *M. binodosa* Pic, 1935; 37. *M. dracuncula* Gressitt, 1942; 38, 42. *M. liubini* sp. nov.; 39, 43. *M. tuberculipennis* Breuning, 1947; 40. *M. castaneoverrucosa* Hayashi, 1974; 27–37, 39, 40. Male; 38. Female. Scale bar 50 μm (41–43), 25–40 not to scale.

Figs 25, 26, 40, Map 1 Chinese common name: 南投小沟胫天牛

Miccolamia castaneoverrucosa Hayashi, 1974: 52. Type locality: Sungkang, Nantou County, Taiwan.
Miccolamia castaneoverrucosa : Hua 2002: 216.
Miccolamia (Miccolamia) castaneoverrucosa : Löbl and Smetana 2010: 224; Lin and Yang 2019: 260; Danilevsky 2020: 315.

#### Type material examined.

***Holotype*** (Fig. 48): • male, “SUNGKANG / TAIWAN / 14.IV.1970 / T.KOBAYASHI”, “HOLOTYPE / *Miccolamia* / *castaneoverrucosa* / Hayashi” [red label] (OSAKA), eight photographs examined provided by Nobuo Ohbayashi.

#### Additional material examined.

Taiwan: 1 male, 1 female, China, Taiwan, Hsinchu, Taiwu, Taikanglintao, 1,850 m, 2014.X.19, leg. Yu-Long Lin (CCCC).

#### Remarks.

Hayashi (1974) differentiated this species from the Japanese *M. verrucosa* Bates by its longer prothorax, which has more distinct lateral and dorsal tubercles or callosities, and by the more strongly uneven elytra with more developed dorsal tubercles and coarser punctures. This species is also similar to *M. tuberculipennis*, but can be separated by the more slender body, lighter coloration of the femora and elytra, and the presence of coarse rugose-punctures on sides of the vertex. Examination of fresh specimens revealed an appendiculate tarsal claw structure, a diagnostic feature not reported in the original description. Lau (2019) recorded this species from Hong Kong. However, the photographic evidence from certain local naturalist websites was later determined to be a misidentification of *Paratimiola rondoni* Breuning, 1965 (Clive Lau pers. comm., 2025).

#### Distribution

**(Map 1).** China: Taiwan.

### ﻿ Miccolamia albosetosa

Taxon classificationAnimaliaColeopteraCerambycidae

Gressitt, 1951

CD4F50DF-D5BD-52F0-9FC9-EDAC125C65C6

Map 1 incertae sedis Chinese common name: 白毛小沟胫天牛

Miccolamia albosetosa Gressitt, 1951: 533. Type locality: Shinsui (=Shenshui, Gaoxiong City), Taiwan.
Miccolamia albosetosa : Breuning 1963: 490; Hua 2002: 216.
Miccolamia (Miccolamia) albosetosa : Breuning 1975: 52, 53; Löbl and Smetana 2010: 224; Lin and Yang 2019: 260; Danilevsky 2020: 315.

#### Original description.

“Subrobusta, postice attenuatis; prothorace breve tuberculatis, haud constrietis; elytris attenuatis, basi subcristatis; corporis parce albo setosis. Long. 3.25 mm.; lat. 1.2.”

Male (?): Dark pitchy brown; reddish on antennae and paler reddish brown on bases of proximal segments; somewhat reddish on antennal supports, tarsi and bases of tibiae. Body irregularly and very thinly clothed with adpressed pale pubescence, and with scattered suberect pale hairs of mediocre length, whitish on sides of body and appendages, brownish on dorsum.

**Figures 44–51.**
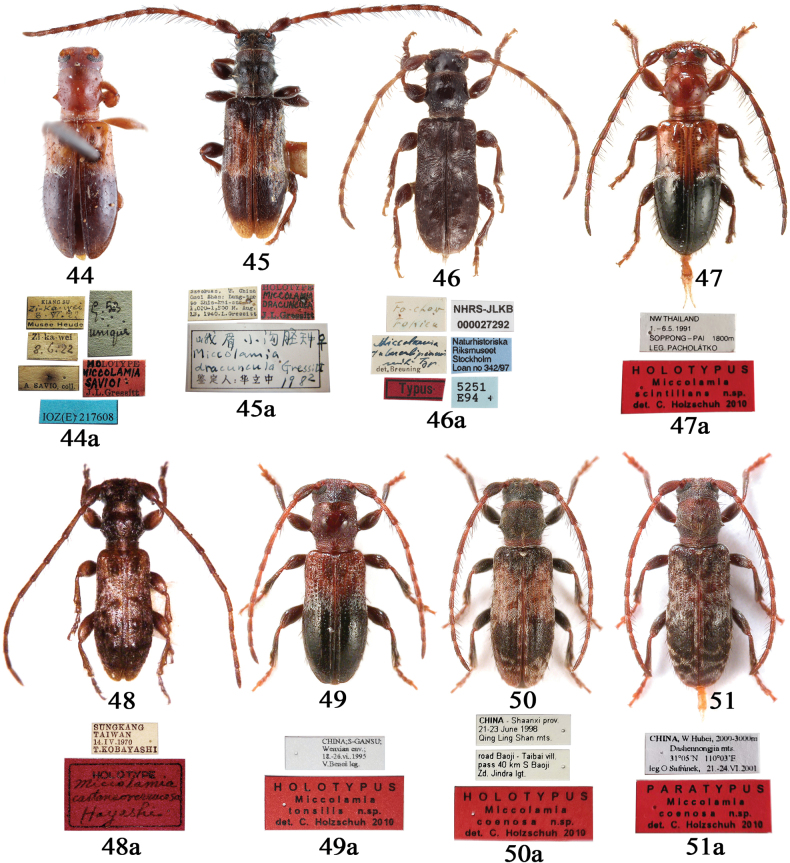
Habitus and labels of the types of *Miccolamia* spp. 44. *M. savioi* Gressitt, 1940 (holotype, female); 45. *M. dracuncula* Gressitt, 1942 (holotype, male); 46. *M. tuberculipennis* Breuning, 1947 (holotype, male, photo Johannes Bergsten, © 2017 NHRS, CC-BY 4.0); 47. *M. scintillans* Holzschuh, 2010 (holotype, female); 48. *M. castaneoverrucosa* Hayashi, 1974 (holotype, male); 49. *M. tonsilis* Holzschuh, 2010 (holotype, male); 50, 51. *M. coenosa* Holzschuh, 2010 (50 holotype, male, 51 paratype, female). a labels.

Head about as broad as prothorax, very finely punctured, feebly concave between antennal insertions; frons nearly twice as wide as high; inferior eye-lobe rounded-triangular, slightly deeper than gena below it. Antennae nearly one-third longer than body, moderately slender; scape feebly swollen, as long as third segment; fourth slightly longer than third; fifth fully as long as third; remainder decreasing slightly. Prothorax subcylindrical, slightly widened and briefly tuberculate at middle of each side. slightly narrowed basally; disc subevenly swollen on central portion, very slightly raised on each side of center, very finely punctured. Scutellum subtriangular. Elytra very slightly narrowed to behind middle, then more strongly narrowed and internally subobliquely truncate; disc of each with a blunt subbasal crest and with fine uneven puncturation, in part arranged in one or two partial rows near center of disc. Ventral surfaces distinctly punctured. Femora clavate.

**Figures 52–60.**
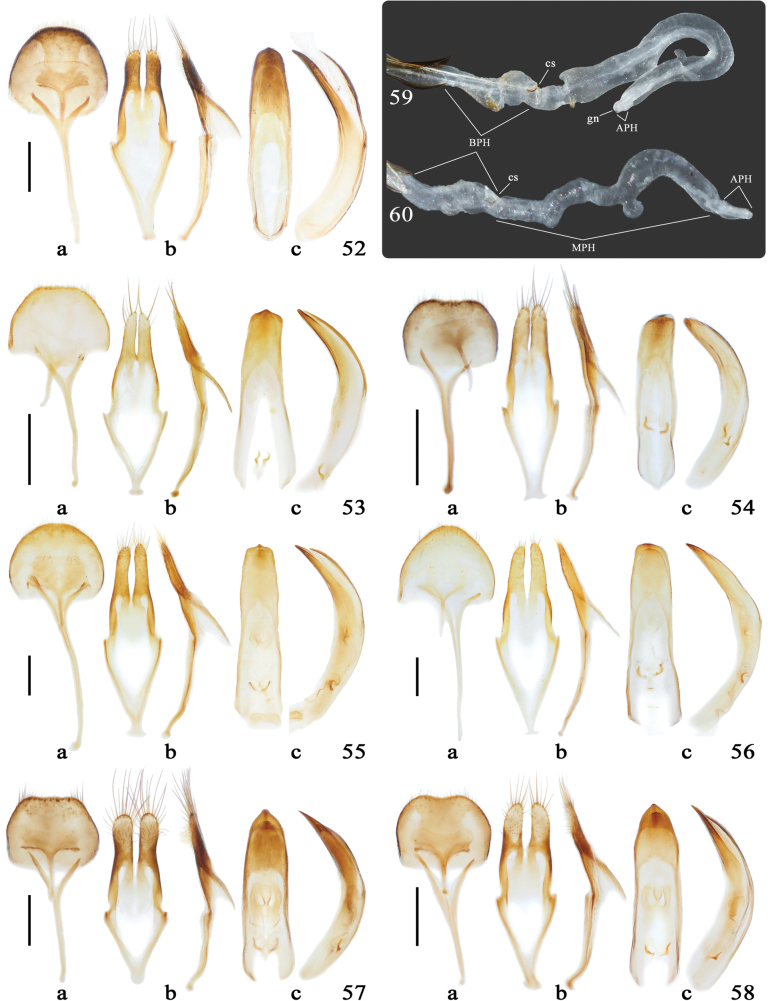
Male terminalia of *Miccolamia* spp. 52–58. Genitalia; 59, 60. Endophallus in inflated and everted condition; 52, 59. *M. savioi* Gressitt, 1940; 53. *M. mystica* sp. nov.; 54, 60. *M. minuta* sp. nov.; 55. *M. yanziae* sp. nov.; 56. *M. holzschuhi* sp. nov.; 57. *M. shennong* sp. nov.; 58. *M. panda* sp. nov. a tergite VIII with sternites VIII and IX in ventral view b tegmen in ventral view and lateral view c median lobe in ventral view and lateral view. Abbreviations: APH apical phallomere BPH basal phallomere MPH median phallomere cs crescent-shaped sclerites gn gonopore (59, 60). Scale bars 0.2 mm (52–58), 59, 60 not to scale.

**Figures 61–66.**
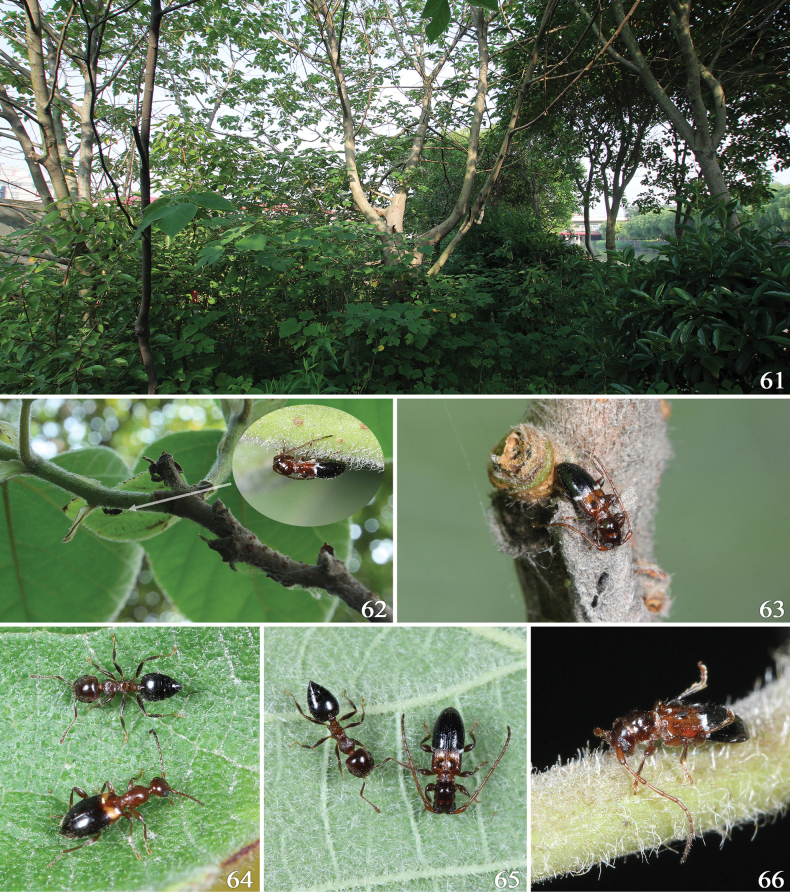
Biotope of *Miccolamia savioi* 61. Habitat with host trees *Broussonetia papyrifera* (Moraceae); 62, 63. Living individuals of *M. savioi* hidden among the twigs; 64, 65. A living individual of Crematogaster cf. zoceensis (Myrmicinae) meets a *Anthelephila mandarina* (Anthicidae) and a *M. savioi* respectively, showing the similarities; 66. A living individual of *M. savioi*, showing a possibly defensive posture.

HOLOTYPE: Male (?) (Taiwan Agric. Res. Inst.), Shinsui, Formosa, March 25, 1940.

#### Remarks.

The holotype of this species could not be located in TARI (Yu-Long Lin pers. comm., 2023) and no additional material was available for this study. According to the original description, this species is characterized by the antennae nearly one-third longer than the body; antennomere III slightly shorter than IV, equal in length to V; pronotum feebly tuberculate laterally and weakly constricted behind the lateral tubercles; pronotal disk very slightly raised on each side of center; elytral apices internally subobliquely truncate; and each elytron bearing only one blunt subbasal tubercle.

According to Gressitt’s data, the holotype exhibits an unusually short body proportion (BL/BW = 2.7), deviating significantly from the range observed in currently studied species (BL/BW = 2.9–3.6). It is unclear whether this represents a genuine morphological difference or a measurement inaccuracy. Furthermore, the distinctive antennal characteristics (proportions of antennomeres III–V combined with exceptional length) show no congruence with any documented *Miccolamia* species (Hasegawa and N. Ohbayashi, 2001; present study, etc.), but instead demonstrate remarkable similarity to certain members of the genus *Rhopaloscelis* Blessig, 1872, such as *R. maculatus* Bates, 1877 from Japan.

Given the absence of critical diagnostic characters in the original description, particularly regarding metatibial morphology, this species is treated here as of uncertain generic position. Further examination of the holotype or topotypes is necessary to confirm its generic placement.

#### Distribution.

China: Taiwan.

##### ﻿Biological notes

Specimens of the genus *Miccolamia* are exceptionally rare in Chinese institution or private collections, with no prior biological data available for Chinese species. This overview primarily derives from the first author’s field observations and limited collector records. Most species inhabit broadleaf deciduous or mixed coniferous and broadleaf forests. Western species (e.g. *M. coenosa*, *M. panda*, *M. shennong*) typically occur at higher elevations (ca 1,500–2,520 m), while some eastern species (*M. minuta*) and widely distributed species (*M. savioi*) occupy lower elevations (ca 0–760 m), including coastal urban forestation zones. Adults are typically collected by beating, sweeping, or direct observation of various hardwood twigs during daytime; exceptions include the holotype of *M. holzschuhi*, which was captured by using a light trap in eastern Yunnan, *M. liubini* from Hainan captured in flight, and *M. binodosa* obtained by canopy fogging in southern Yunnan.

The genus appears polyphagous: *M. tuberculipennis* utilizes recently dead twigs of *Platycarya strobilacea* Sieb. et Zucc. (Juglandaceae) and *Lindera* sp. (Lauraceae) in Zhejiang; *M. coenosa* and *M. tonsilis* associate with *Cornus* spp. (Cornaceae) in Shaanxi, with the latter species also recorded on *Broussonetia papyrifera* (Linn.) (Moraceae) in Hubei. *Miccolamia minuta* occurs on *B. papyrifera*, *Maclura tricuspidata* (Carr.) (Moraceae) in Anhui, and *Styrax odoratissimus* Champ. (Styracaceae) in Zhejiang. Three *M. dracuncula* adults emerged from *Daphniphyllum macropodum* Miq. (Daphniphyllaceae) twigs in Sichuan, while others were beaten from *Cornus* spp. Host records remain incomplete for some species, e.g., *M. shennong* is known only from *Sorbaria arborea* Schneid. (Rosaceae), and *M. yanziae* from an unidentified oak in Xizang. Sympatry occurs occasionally and is generally limited to two species, with an exceptional case recorded in Hubei where three species, *M. savioi*, *M. tonsilis*, and *M. mystica*, were observed co-existing on the same *B. papyrifera* tree.

Detailed observations of *M. savioi* from Shanghai and Zhejiang reveal adults active May to July in lowland deciduous forests and urban wastelands (e.g., Fig. 61, a key Shanghai site at 31°07'51"N, 121°23'26"E, which was since destroyed by garden projects during 2021). Primary hosts include *B. papyrifera* and occasionally *Morus alba* Linn. (Moraceae), with three records from *Broussonetia kazinoki*. Adults frequent shaded microhabitats, clinging to leaf undersides or dark shoots (Figs 62, 63). Larvae tunnel in recently dead *B. papyrifera* twigs (~4–5 mm diameter) on living trees, completing life cycle in one year. Distinctive larval morphology (acute abdominal spinule on tergite IX) facilitates identification (Tsherepanov 1991). Adults exhibit ant-mimicry resembling co-occurring ants, Crematogaster cf. zoceensis Santschi, 1925 (Myrmicinae) (Fig. 65), and ant-like flower beetles, *Anthelephila mandarina* (Boheman, 1858) (Anthicidae) (Fig. 64). Unique possibly defensive behaviors include twig-clasping with antennae while rapidly alternating hind leg movements (Fig. 66). The metatibial morphology (i.e., deep subapical sinus lined with dense long setae) functions as a leg-cleaning structure, likely co-occurring across the genus.

## Supplementary Material

XML Treatment for Miccolamia

XML Treatment for Miccolamia savioi

XML Treatment for Miccolamia mystica

XML Treatment for Miccolamia minuta

XML Treatment for Miccolamia scintillans

XML Treatment for Miccolamia yanziae

XML Treatment for Miccolamia holzschuhi

XML Treatment for Miccolamia tonsilis

XML Treatment for Miccolamia coenosa

XML Treatment for Miccolamia shennong

XML Treatment for Miccolamia panda

XML Treatment for Miccolamia binodosa

XML Treatment for Miccolamia dracuncula dracuncula

XML Treatment for Miccolamia dracuncula orientalis

XML Treatment for Miccolamia liubini

XML Treatment for Miccolamia tuberculipennis

XML Treatment for Miccolamia castaneoverrucosa

XML Treatment for Miccolamia albosetosa

## ﻿Additional information

### Use of AI

No use of AI was reported.

### Author ORCIDs

Wen-Xuan Bi https://orcid.org/0000-0003-1806-7679

Chang-Chin Chen https://orcid.org/0000-0001-5771-8337

Mei-Ying Lin https://orcid.org/0000-0001-9219-724X

### Data availability

All of the data that support the findings of this study are available in the main text
